# Pressure Sensing and Kinetic Modeling of Oxygen-Releasing Endoperoxides for Chemically Driven Micro-Actuation

**DOI:** 10.1021/acsomega.5c07255

**Published:** 2025-09-24

**Authors:** Abhishek Sharma, Vanessa Barth, Henning J. Jessen, Laura M. Comella

**Affiliations:** † Cluster of Excellence livMatS @ FIT-Freiburg Center for Interactive Materials and Bioinspired Technologies, 9174 University of Freiburg, 79110 Freiburg, Germany; ‡ Laboratory for the Design of Microsystems, Department of Microsystems Engineering (IMTEK), University of Freiburg, 79110 Freiburg, Germany; § Institute of Organic Chemistry, University of Freiburg, 79104 Freiburg, Germany; ∥ Institute of Energy Efficient Mobility, 38992 Karlsruhe University of Applied Sciences, 76133 Karlsruhe, Germany

## Abstract

This work presents a pressure-based sensing framework to directly track and monitor oxygen release from anthracene-endoperoxide (ANT-EPO) decomposition in an aqueous environment. ANT-EPO shows potential as an on-demand gas source for applications in pneumatic actuators and soft robotics. However, its integration into functional systems requires accurate, real-time characterization of oxygen release kinetics. To address this, a sealed measurement chamber coupled with a calibrated low-pressure sensor was employed to enable long-term, continuous tracking of oxygen release under ambient conditions (37 °C) and at room temperature. A multistep signal processing pipeline was implemented to extract meaningful kinetic information from the pressure response resulting from oxygen release. The raw pressure–time signals were processed using an adaptive Savitzky–Golay (SG) smoothing followed by discrete wavelet transform (DWT) to recover clean reaction kinetics that were further analyzed using kinetic modeling. The denoised pressure data revealed temperature-sensitive decomposition kinetics, with room temperature experiments exhibiting a sigmoidal profile, well captured by a logistic model (*R* ^2^ = 0.993, RMSE = 0.17 kPa), outperforming a first-order model in capturing this multiphase behavior. In contrast, under constant 37 °C conditions, the pressure rise followed a first-order kinetic profile (*R* ^2^ = 0.995), reflecting accelerated and linear oxygen release kinetics. These findings demonstrate that pressure sensing can serve as a standalone, real-time, and quantitative characterization of oxygen release kinetics, even for slow-reacting systems. This methodology establishes a structured sensing framework combined with a multistage processing pipeline for tracking gas release dynamics and capturing dynamic deviations from ideal kinetics. This study lays the groundwork for pressure-driven actuator designs and advanced feedback control in soft robotic systems, highlighting the broader impact of oxygen-releasing compounds.

## Graphical abstract

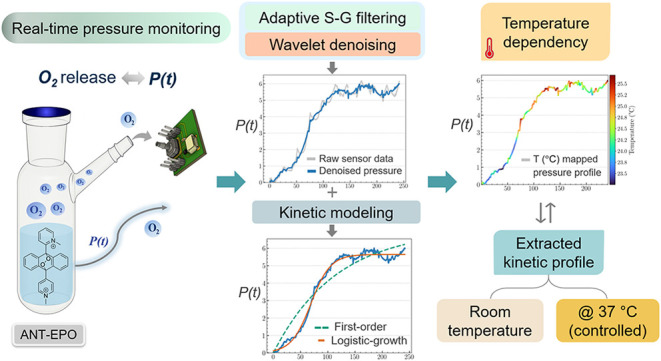

## 1 Introduction

The controlled release of gases from chemically triggered responsive materials offers a promising mechanism for actuating soft robots and pneumatic platforms.^1–3^ Gas released from responsive materials generates on-demand pressure, thus enabling untethered motion and actuation by converting chemical energy directly into mechanical motion.^4,5^ Among these, oxygen-releasing compounds such as anthracene-endoperoxides (ANT-EPO) are of particular interest due to their ability to reversibly bind oxygen and release it upon decomposition in aqueous environments. This unique capability positions ANT-EPO as a promising candidate for pressure-driven actuation and self-regulating oxygen delivery systems. In addition, oxygen-releasing compounds can be used to release singlet oxygen or sustain cells under anoxic conditions.^6–8^ Singlet oxygen release and photochemical afterglow have also been used for information encryption and bioimaging.^7,9,10^ However, effectively utilizing such gas-releasing compounds requires a detailed understanding of their pressure dynamics, particularly in slow-release systems where pressure changes are subtle and occur over extended durations.

While ANT-EPO has been widely studied in the context of singlet oxygen delivery in photodynamic therapy (PDT)^11–13^ and in optical sensing,^14^ its potential as a direct pressure-generating reagent for actuation has not yet been characterized. Quantitative tracking of the pressure response during ANT-EPO decomposition is an important step for designing adaptive systems that rely on chemical-to-mechanical energy conversion. This work investigates the pressure profiles generated from the ANT-EPO decomposition in a sealed environment, with the goal of establishing a kinetic profile that supports its integration into chemically driven actuation. ANT-EPO decomposes in water to form anthracene (ANT) and releases molecular oxygen in a defined 1:1 stoichiometric ratio,^7,8^ offering a predicable pathway to track the oxygen output. The decomposition rate and oxygen release kinetics from ANT-EPO are sensitive to temperature and molecular structure, resulting in tunable half-lives. The clear stoichiometry of oxygen release makes ANT-EPO an attractive source for programmable on-demand oxygen generation.^9,15–17^

Existing oxygen sensing methods such as optical absorbance,^14,18,19^ fluorescence quenching,^20–22^ solid-state sensors,^23,24^ and electrochemical oxygen sensors^25^ are optimized for detecting dissolved oxygen concentration in solutions.^26^ Despite their potential, these techniques are not well suited for continuous monitoring of slow, cumulative gas-releasing reactions. These methods often rely on indirect measurement, are limited by solution saturation, and typically require clear optical pathways.^23^ These constraints motivate the exploration of complementary sensing strategies that can overcome optical interference and directly track dynamic gas evolution. Several studies on gas evolution from chemical reactions in closed systems,^27–30^ highlight the potential of gas release for microrobotic propulsion and reaction tracking. However, no prior study has quantitatively tracked pressure build-up from ANT-EPO decomposition. By directly transducing oxygen evolution into measurable pressure signal, a complete time-resolved monitoring of the reaction progress can be achieved, offering unique insights into the kinetics of slow gas-releasing processes.

The proposed work in this paper focuses on characterizing the oxygen release kinetics of ANT-EPO decomposition through pressure-based monitoring combined with a tailored multistage signal processing pipeline to extract meaningful kinetic information. This integrated framework enables continuous time-resolved monitoring of pressure signals with signal enhancement techniques. Savitzky–Golay (SG) smoothing was first applied to reduce high-frequency noise while preserving the true signal’s features,^31^ followed by discrete wavelet transform (DWT) filtering to isolate the pressure trend associated with oxygen release. The resulting denoised pressure–time profile was further analyzed using both first-order and logistic kinetic models to characterize the overall reaction behavior and extent of oxygen release. This sensing and modeling framework provides insights into how pressure build-up can serve as a functional readout of chemical reaction progress, linking chemical decomposition to mechanical energy output.

This work provides the first direct pressure-based quantification of oxygen release from ANT-EPO, offering new insights into their gas-generating behavior and potential for integration into soft robotics or pneumatic actuation systems. The following sections detail the methodology and experimental setup, with the results and discussion on how ANT-EPO’s decomposition behavior was elucidated through this pressure-sensing approach. By providing a direct quantitative interpretation of oxygen releasing dynamics, this work establishes a clear, standalone contribution toward the broader use of ANT-EPO in chemically driven actuation platforms requiring on-demand gas generation.

## 2 Experimental Section

### 2.1 Chemical and Reagents

Water-soluble anthracene-endoperoxide (ANT-EPO) was used as the oxygen-releasing compound due to its clean decomposition reported in literature.^8^ ANT-EPO was synthesized following established protocols, with specific modifications adapted for this study. A detailed description of the modified synthesis procedure is provided in the Supporting Information (SI, 1.1). Upon dissolution in deionized (DI) water, ANT-EPO decomposes into anthracene (ANT), and molecular oxygen in a 1:1 stoichiometric ratio.^7,32^ This study focuses on the forward decomposition process of ANT-EPO, with half-lives of 35.8 h at room temperature (∼24 °C) and 4.7 h at 37 °C.

### 2.2 Pressure-Sensing Setup

The experimental setup consisted of a Schlenk tube (FengTecEx, Germany) sealed with a silicone tube and Luer-lock connector (neoLab, Germany), connected to a custom sensor module. The module included a digital gauge pressure sensor (0–16 kPa, ABP2LANT160MGSA3, Honeywell, USA) and a digital temperature–humidity sensor (SHT45, Sensirion, Switzerland), both interfaced with a low-power microcontroller (XIAO nRF52840, seeed studio) operating at 1 Hz sampling rate. Real-time data were displayed via a 0.96” OLED display (SSD1315, seeed studio) and simultaneously logged using a background script managed via the nonsucking service manager (NSSM) on the host system. This configuration enabled continuous data acquisition over multiple half-lives of ANT-EPO with minimal manual intervention. Measurements were conducted at both room temperature and 37 °C in a temperature-controlled chamber. Figure 1 illustrates the schematic of the experimental setup.

**Figure 1:**
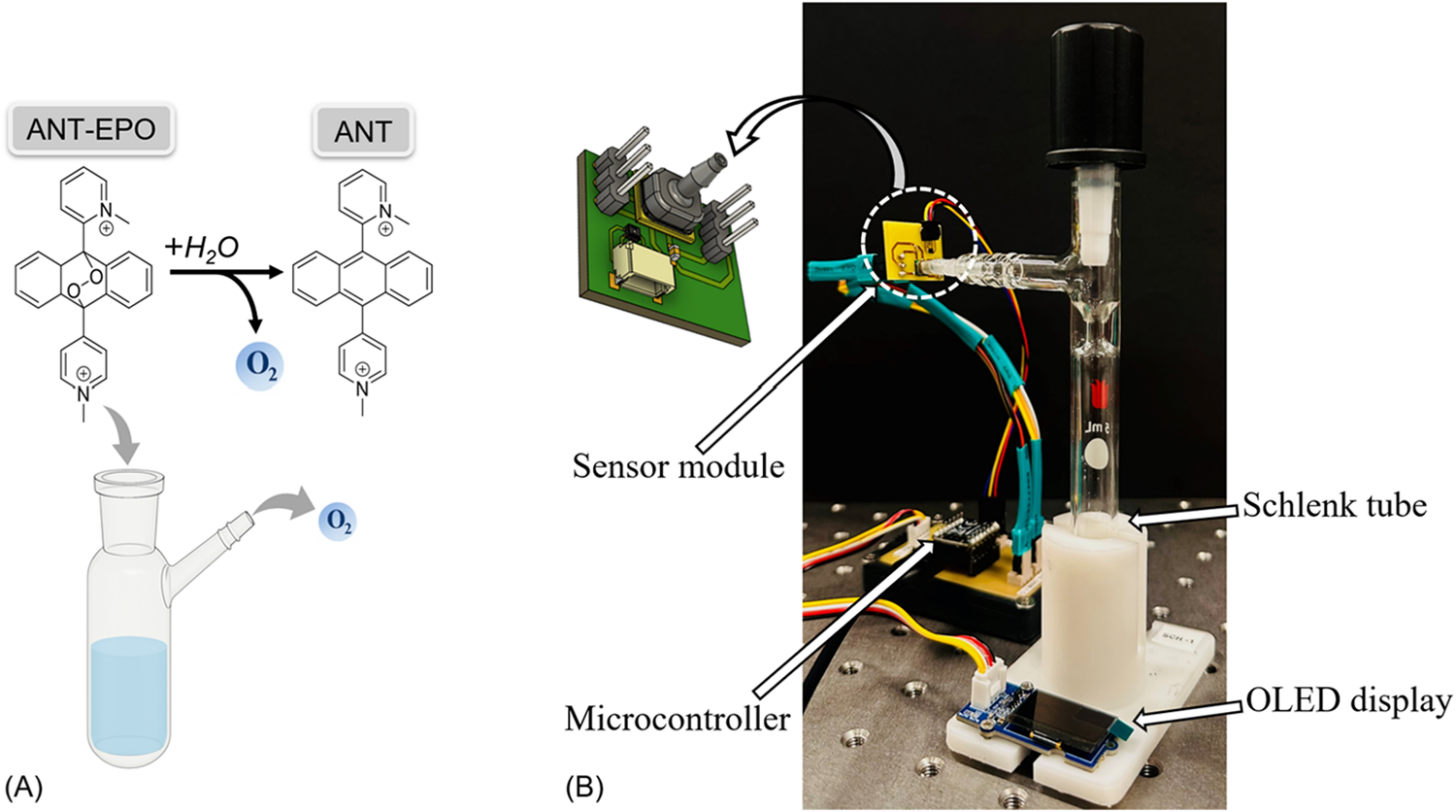
(A) Decomposition reaction of anthracene-endoperoxide (ANT-EPO) to anthracene (ANT) in an aqueous environment with the release of molecular oxygen. (B) Experimental setup for pressure-based monitoring of oxygen release from ANT-EPO decomposition. A sealed Schlenk tube was connected to a low-pressure (ABP2) and temperature (SHT45) sensor module, interfaced with a microcontroller and OLED for real-time data acquisition and display. The inset illustrates the schematic of the sensor module.

### 2.3 Signal Processing and Kinetic Modeling

To interpret the raw pressure–time profiles and extract meaningful kinetic parameters, a structured signal processing pipeline was applied to suppress noise, correct drift, and recover the true oxygen-induced pressure response (Figure 2). Each processing step was selected based on its methodological fit to the signal characteristics and was empirically validated using performance metrics.

**Figure 2:**
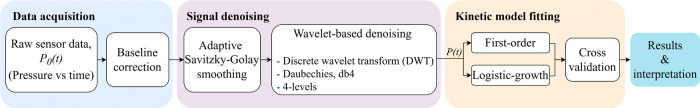
Signal processing workflow used to extract oxygen release pressure profiles from raw pressure measurements. Analysis steps includes, baseline correction, adaptive Savitzky–Golay smoothing, and discrete wavelet transform (DWT) filtering, followed by kinetic model fitting.

#### 2.3.1 Baseline Correction and Adaptive Smoothing

The raw pressure signals were initially corrected using baseline references from control measurements to eliminate static offsets and drift unrelated to ANT-EPO’s decomposition. The corrected signal was subsequently smoothed using a Savitzky–Golay (SG) filter to reduce high-frequency noise while preserving the key kinetic features of the signal profile such as curvature and inflection regions of the reaction.^31,33^ The SG filter was chosen over moving average and Gaussian filters due to its ability to retain local polynomial trends without flattening signal transitions and distorting early stage signal onset or saturation behavior.

Rather than applying an arbitrary window size to the SG filter, an adaptive window selection strategy was employed to optimize the filter performance.^34,35^ The optimal window configuration was selected based on a scoring function derived from signal-to-noise ratio (SNR) improvement and root-mean-square error (RMSE) minimization between raw and filtered signals (see SI, Table S1 for scoring equation and parameter weights). This strategy allowed robust smoothing without distorting the underlying pressure signal.

#### 2.3.2 Wavelet-Based Drift Removal

The SG smoothed signal was further processed using a discrete wavelet transform (DWT) employing Daubechies 4 (db4) wavelets to isolate the slow varying drifts arising due to sensor offset or thermal expansion effects over extended durations. DWT enables multiresolution decomposition of the underlying pressure signal into frequency-specific components. The smoothed signal was decomposed into multiple frequency bands as detail coefficients (D3-D5),^33,36,37^ which correspond to midfrequency components capturing the active release process. The low-level approximation coefficient (A5), which captured long-term drift and baseline instability, was excluded during signal reconstruction. These selected midfrequency detail levels were recombined to yield a denoised and drift-corrected pressure signal, *P­(t)*, suitable for kinetic model fitting. This step enabled clear separation of the pressure dynamics caused by ANT-EPO decomposition from background drift trends (see SI Figures S23 and S24 for DWT components selection).

#### 2.3.3 Kinetic Model Fitting

The denoised pressure signal, *P­(t)*, was fitted using two kinetic models, a first-order exponential^38^ and a logistic-growth model.^39^ The first-order model assumes concentration dependent decomposition with a constant rate, while logistic model captures sigmoidal trend, including an initial lag phase, accelerated growth, and eventual saturation phase. Model fitting was performed using constrained nonlinear least-squares optimization, to ensure stable and physically meaningful solutions.^40,41^ Full model equations and parameter bounds are included in SI, Table S2.

#### 2.3.4 Temperature Dependence and Model Evaluation

To assess the effect of temperature on ANT-EPO’s reaction kinetics, model fits were applied to the pressure data recorded at room temperature and at elevated temperature (∼37 °C) in a controlled chamber. Furthermore, Arrhenius-type analysis was applied on the pressure measurements,^42,43^ *P­(t,T)*, by extracting rate constants, *k­(T)*, from the fits and plotting their temperature dependence to validate the expected thermally accelerated decomposition behavior (detailed in SI Table S2). Additionally, pressure sensor behavior across the temperature range was independently evaluated using reference measurements to confirm the sensor’s thermal stability. This ensured the temperature effects observed in ANT-EPO decomposition reflects true oxygen release rather than sensor bias.

Model performance was evaluated using standard metrics including coefficient of determination (*R* ^*2*^), root-mean-square error (RMSE), and Akaike information criterion (AIC) with lower AIC values indicating better model fit.

### 2.4 Sample Preparation and Measurement

ANT-EPO samples were dissolved in deionized (DI) water and transferred into a sealed Schlenk tube. The sensor module was mounted to the tube’s headspace, and continuous data logging was initiated immediately. To capture the complete decomposition process, measurements were conducted over durations exceeding seven half-lives of ANT-EPO at the respective temperature (room temperature and 37 °C). This period ensured more than 99% conversion of ANT-EPO to ANT and resulting oxygen release.

Two ANT-EPO samples were analyzed, sample M_1_ (2.5 mM) was measured at room temperature, while sample M_2_ (2.18 mM) was tested both at room temperature and in parallel at 37 °C in a temperature-controlled chamber. These concentrations were selected to ensure measurable oxygen evolution in the headspace within the resolution limits of the pressure sensor. The resulting pressure–time profiles were processed using the signal processing pipeline and kinetic modeling workflow described in Section 2.3, to extract kinetic parameters and features such as reaction onset, inflection regions, maximum pressure, and saturation behavior.

## 3 Results and Discussion

### 3.1 Pressure–Time Response of ANT-EPO Decomposition

Pressure–time measurements were performed for samples M_1_ and M_2_ to characterize the complete oxygen release behavior of ANT-EPO over extended periods (>250 h) at room temperature and under a controlled temperature setting. The pressure profiles obtained after the signal processing pipeline steps mentioned in Section 2.3 are shown in Figure 3, providing comparison with the raw signal. Both concentrations M_1_ (Figure 3(a)) and M_2_ (Figure 3(b)) exhibited a characteristic sigmoidal trend consistent with oxygen release from ANT-EPO decomposition at room temperature. An initial lag phase was followed by a pressure rise and eventual saturation, indicating molecular oxygen release and completion of the reaction.

**Figure 3:**
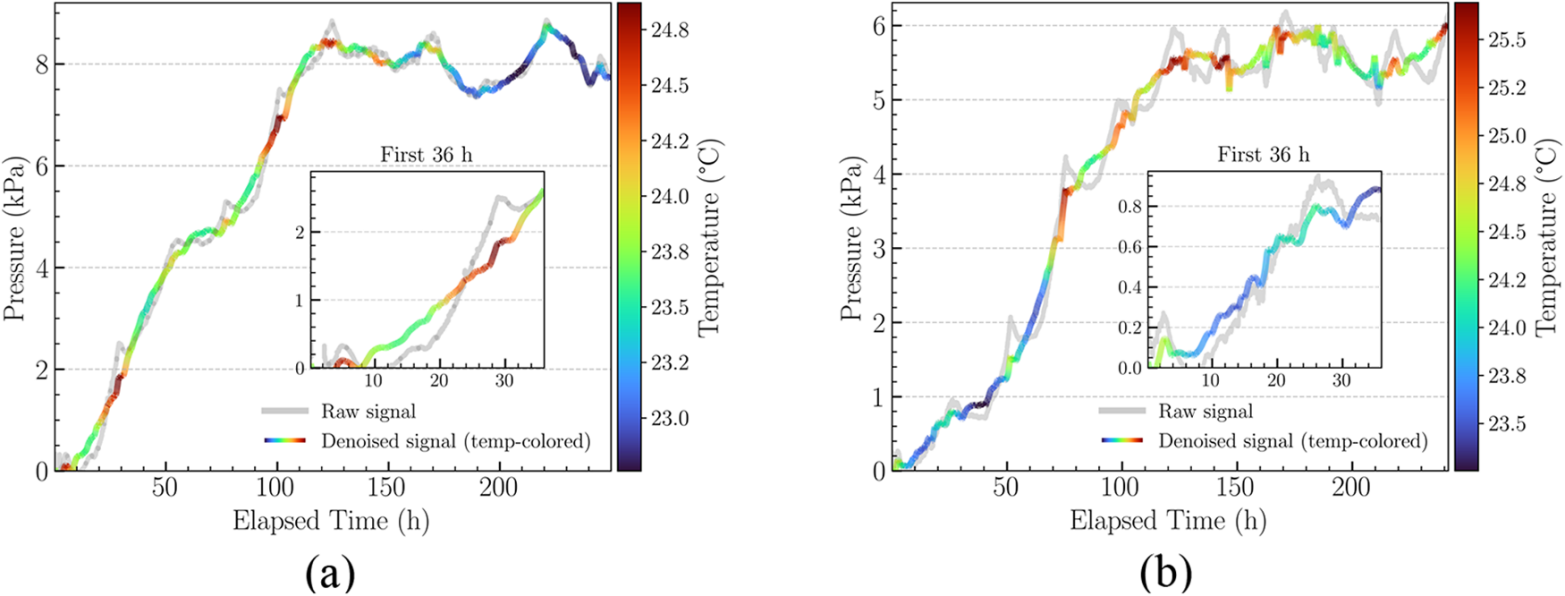
Pressure–time curves with ambient temperature colormap overlays. (a) M_1_, (b) M_2_. Raw data (gray) are shown together with the denoised pressure curves overlaid with ambient temperature colormaps. Warmer intervals (green to red) correspond to slightly faster pressure increase, highlighting the effect of subtle thermal fluctuations on the reaction kinetics.

The signal processing steps enabled clear visualization of low-amplitude pressure changes during the early reaction phases and improved separation of kinetic features from background drift. The reconstructed pressure signals preserved the onset, growth, and saturation regions of the reaction with high fidelity. Filtering parameter metrics and detailed signal decomposition are provided in the SI (Figures S19–S24).

To evaluate the impact of ambient temperature fluctuations on the reaction kinetics, pressure–time plots for M_1_ and M_2_ at room temperature were overlaid with temperature colormaps (Figure 3). Slight pressure shifts corresponded to warmer periods (green-to-red regions), while cooler periods (blue gradient) coincided with transient slowdowns. These subtle variations (0.1–0.2 kPa) were consistent with minor ambient temperature fluctuations (22.8–24.2 °C) and highlight the importance of temperature tracking in interpreting low-amplitude pressure measurements.

In comparing M_1_ and M_2_, the maximum pressure reached at room temperature was ∼8.2 kPa and ∼5.8 kPa, respectively, resulting in a ratio of ∼1.4. This was larger than the concentration ratio (2.5/2.18 ≈ 1.15), indicating that the pressure signal does not scale linearly with ANT-EPO concentration. Possible contributing factors include (i) the nonlinear relationship between dissolved oxygen solubility and headspace partitioning under near-saturation conditions, (ii) experimental variations in the headspace-to-solution ratio that can change apparent pressure differences, and (iii) the influence of temperature-driven variations on the decomposition kinetics and oxygen release dynamics. While these factors provide a plausible explanation, the precise concentration dependence of pressure evolution remains a subject for further investigation.

Sample M_2_, tested additionally at around 37 °C under controlled thermal conditions (Figure 4), demonstrated a significantly shorter induction period and a faster pressure increase, consistent with the decomposition kinetics of ANT-EPO at elevated temperature.^8^ Due to the controlled thermal conditions, a single pass SG smoothing was sufficient to preserve the kinetic signal profile of M_2_. Moreover, at 37 °C, temperature colormap was not required, as the thermal chamber maintained a stable temperature throughout the experiment.

**Figure 4:**
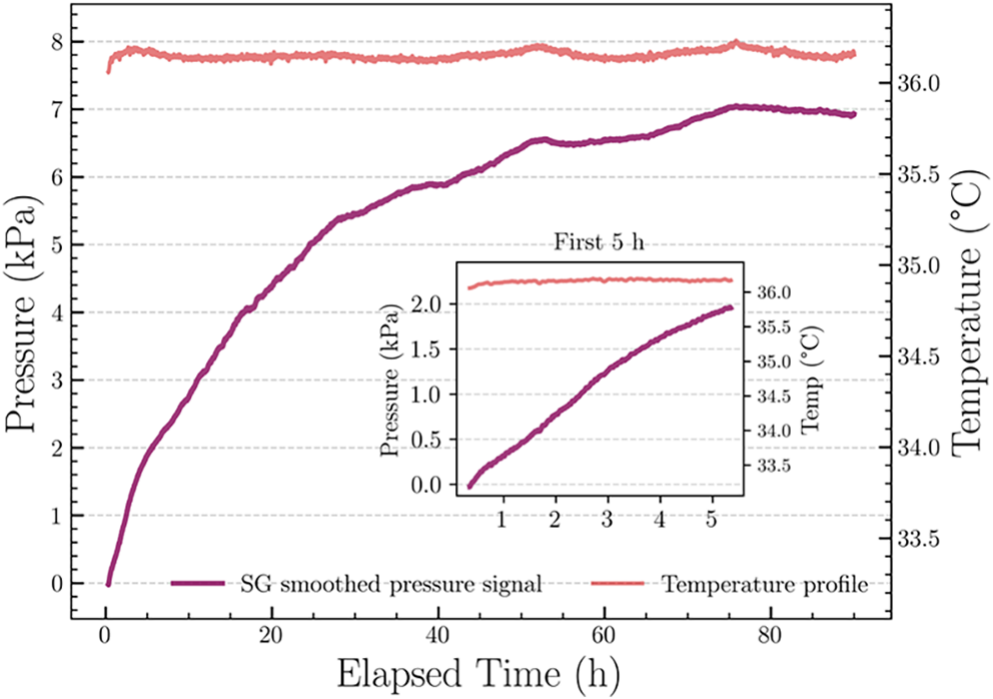
Smoothed pressure–time profile of sample M_2_ at 37 °C in a controlled thermal chamber. Savitzky–Golay (SG) smoothed pressure–time curve showing a rapid pressure rise and early saturation within ∼40 h, with minimal external fluctuation.

Across all experiments, the postsaturation pressure remained stable over time, indicating minimal change and complete ANT-EPO decomposition. The consistency and reproducibility of the pressure trends confirm the reliability of the pressure-sensing approach and signal processing pipeline. The observed pressure trend closely matched the expected stoichiometric oxygen release, further validating ANT-EPO as a reliable candidate for oxygen delivery. The agreement between the expected stoichiometry and the measured pressure kinetics demonstrates that the sensing approach provides dynamic validation of oxygen release in closed systems. Future work may incorporate complementary techniques such as gas chromatography in larger-volume or flow through setups to broaden the characterization of oxygen release from ANT-EPO.

### 3.2 Kinetic Model Fitting and Temperature Dependence

Samples M_1_ and M_2_, measured at room temperature exhibited sigmoidal pressure–time trends, indicating that the decomposition reaction follows a complex, multiphase progression instead of a simple first-order decay. This observed behavior likely results from a combination of phase-dependent kinetics, solvent saturation and minor ambient temperature fluctuations (as discussed in Section 3.1). A more flexible modeling was required to accurately capture the full kinetic trend, particularly around the inflection and saturation phases. Model selection was based on the characteristics of the underlying data and experimental conditions of ANT-EPO decomposition process.

The denoised pressure–time profiles of the samples were fitted using both first-order and logistic kinetic models to characterize the kinetics of oxygen release. The first-order model captured the general rise in pressure but slightly overshoot at the curvature near the inflection region and saturation plateau. In contrast, the logistic model provided a better fit across the entire reaction phases, particularly in capturing the lag phase and saturation (Figure 5). Models fitting results are summarized in Table 1. Across all samples, the logistic model consistently yielded better fit quality, as indicated by higher *R* ^2^, lower RMSE. For instance, in sample M_2_ at room temperature, the logistic model yielded an *R* ^2^ of 0.993 and RMSE of 0.19 kPa, outperforming the first-order model (*R* ^2^ = 0.908, RMSE = 0.29 kPa). Additionally, lower AIC values also favored the logistic fit, confirming its suitability for describing the complex kinetics of ANT-EPO decomposition.

**Figure 5:**
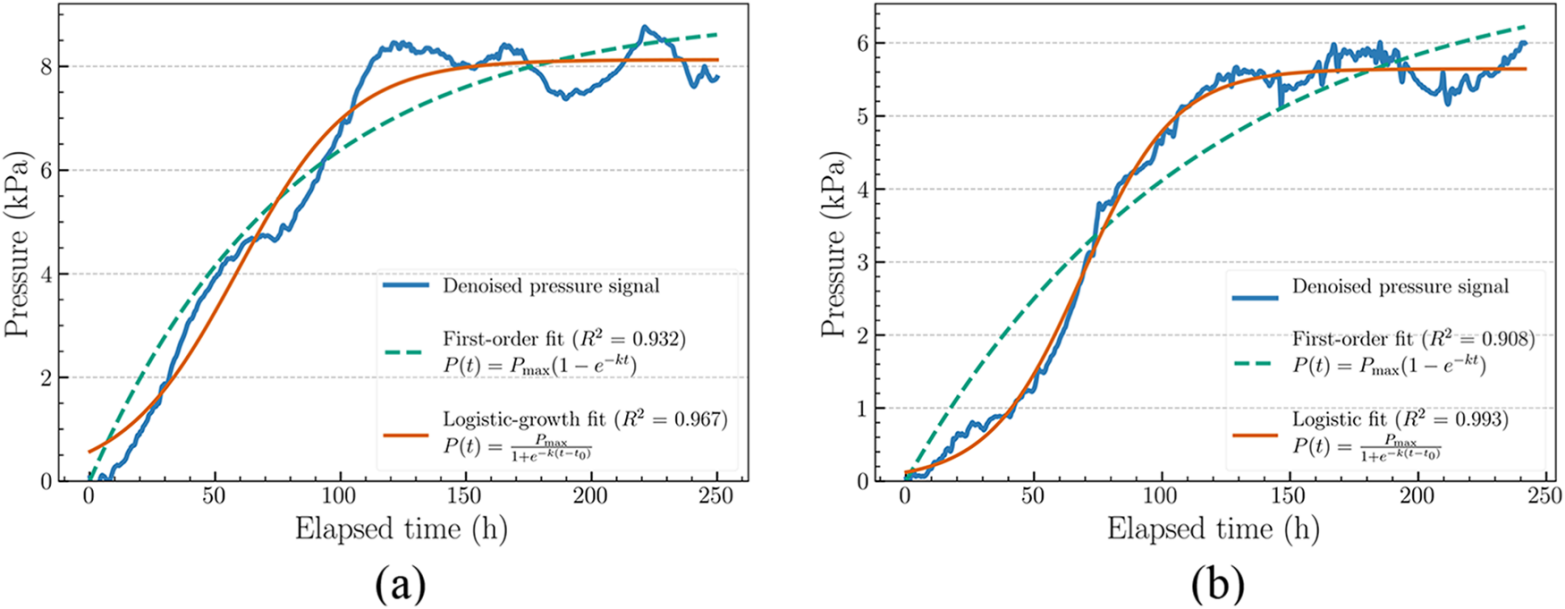
Kinetic model fits for ANT-EPO decomposition at room temperature: (a) M_1_ and (b) M_2_. First-order model fit (dashed) underestimates the lag and saturation regions, while the logistic model (solid) provides better overall fit, especially in the inflection zone.

**Table 1:** Comparison of Kinetic Model Fitting Parameters (Coefficient of Determination, *R* ^2^; Root-Mean-Square Error, RMSE; Akaike Information Criterion, AIC) across Tested Samples of Anthracene-Endoperoxide (ANT-EPO) at Different Temperatures

sample	*T* (°C)	model	RMSE (kPa)	AIC	*R* ^2^
M_1_	*RT*	logistic	0.478	–1.32 × 10^6^	0.967
		first-order	0.689	–6.7e × 10^6^	0.932
M_2_	*RT*	logistic	0.174	–3.03e × 10^6^	0.993
		first-order	0.618	–8.36e × 10^6^	0.908
M_2_	37	first-order	0.115	–1.39e × 10^6^	0.995

To investigate thermal effects, sample M_2_ was measured under controlled thermal conditions at 37 °C in a temperature-stabilized chamber (Figure 6). The elevated temperature significantly reduced the induction period and led to faster saturation, with oxygen release completed and plateau reached within ∼30 h as compared to ∼160 h at room temperature. This behavior was consistent with the known temperature dependence of ANT-EPO decomposition, where half-life reduce from 35.8 h at 24 °C to 4.7 h at 37 °C. Notably, the final pressure plateau remained stable across both temperatures, confirming complete oxygen release despite the faster kinetics.

**Figure 6:**
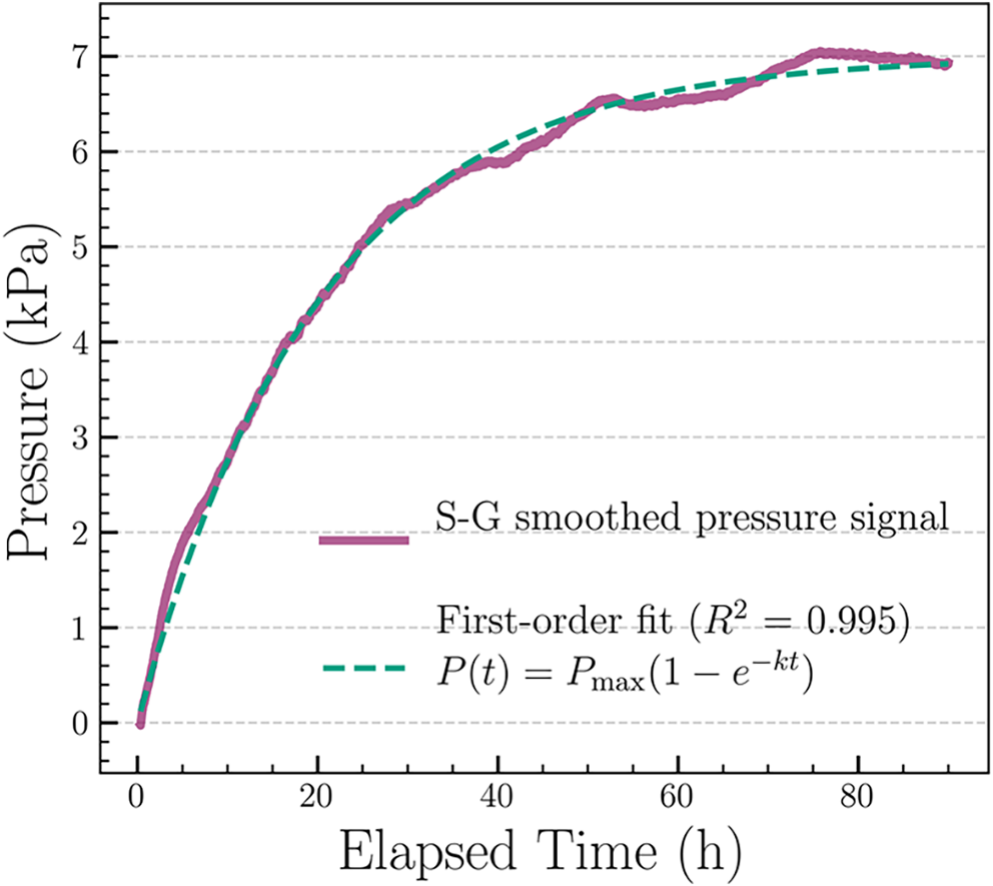
Kinetic model fitting for Sample M_2_ at 37 °C. A rapid decomposition profile fitted by a first-order model under isothermal conditions.

To further assess the temperature dependence of ANT-EPO decomposition, rate constants (*k­(T)*) were extracted from the kinetic model fits of pressure data from samples M_1_ and M_2_ at room temperatures. These were used to construct an Arrhenius-based plot (Figure 7). The linearity of the Arrhenius plots confirmed the expected exponential relationship between reaction rate and temperature. The first-order fit quality slightly improved and the logistic-growth model retained its characteristic sigmoidal shape (see SI Table S3 for model fit parameters). This analysis supports literature-reported thermal behavior of ANT-EPO and provides a predictive basis for estimating oxygen release rates under different thermal conditions. Furthermore, the similarity in final pressure plateaus under both concentrations M_1_ and M_2_ confirms that the observed rate differences stem from true kinetic changes rather than sensor artifacts, supporting the stability of the sensing approach.

**Figure 7:**
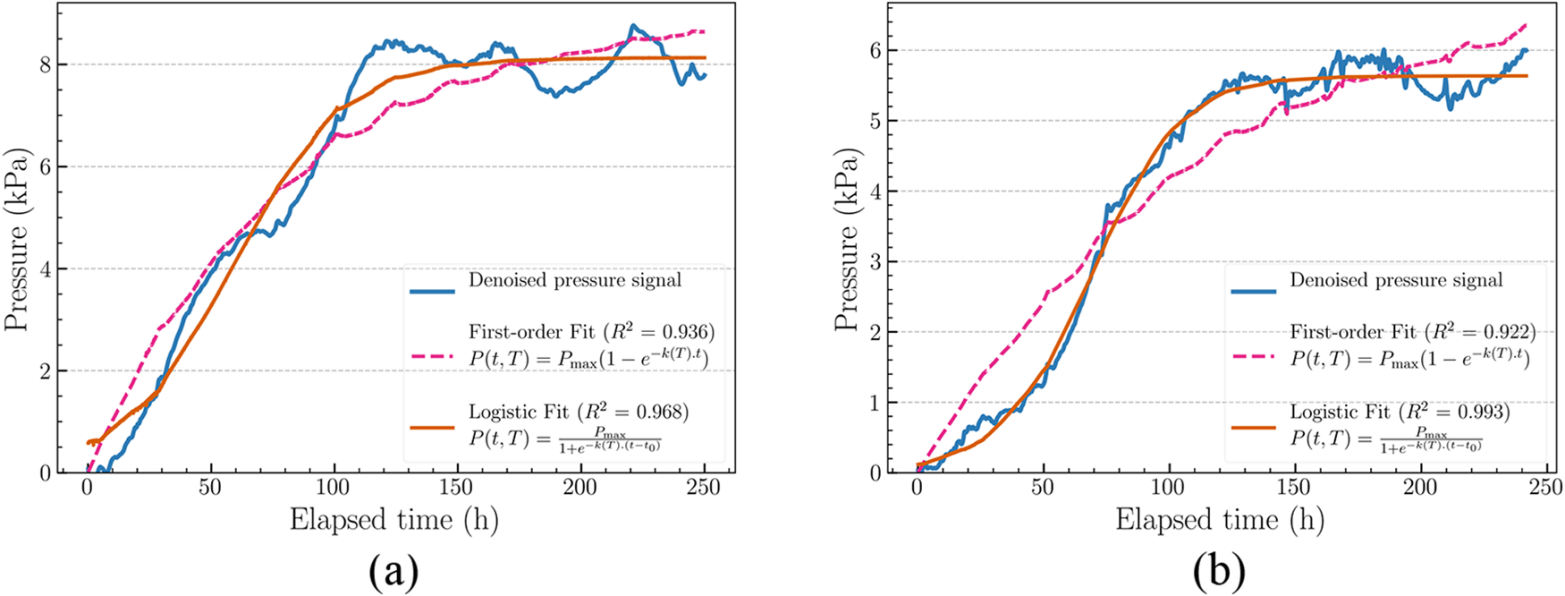
Arrhenius plots using extracted fitted rate constants for (a) M_1_ and (b) M_2_. Linear fit confirms temperature dependence of ANT-EPO’s decomposition kinetics.

## 4 Conclusion

This work establishes a real-time pressure-based sensing and multistep signal processing framework for characterizing the oxygen release kinetics from anthracene-endoperoxide (ANT-EPO) molecules under both ambient and elevated temperature conditions. By combining adaptive Savitzky–Golay smoothing and wavelet-based denoising, this method effectively isolates the relevant kinetic pressure response reflecting true oxygen release from the low-amplitude noise and drift. Room temperature experiments revealed sigmoidal pressure–time behavior consistent with a multiphase behavior, including induction, growth, and saturation phases. These dynamics were well described by a logistic-growth model, which outperformed the first-order model in capturing the nonlinear nature of the decomposition process. In contrast, under controlled 37 °C conditions, the pressure signals followed a first-order profile, indicating accelerated and more uniform release behavior. These findings provide a reliable basis for quantitative evaluation of slow oxygen-releasing systems in sealed environments. This sensing strategy is broadly applicable to other cumulative gas-releasing processes, where direct oxygen quantification is hindered by saturation effects or optical constraints. By providing a direct and quantitative characterization of pressure evolution from an oxygen-releasing reaction, this work establishes a reference framework for the development of responsive material systems and autonomous flexible devices.

## Supplementary Material



## Notes

Chemical synthesis pathway of ANT-EPO (Figure S1); reaction schemes of compounds 2–6 (Figures S2–S6); NMR spectra of compounds 2–6 (Figures S7–S18); measurement analysis plots, filtering parameter metrics, and signal decomposition (Figures S19–24); adaptive Savitzky–Golay filter evaluation and scoring function (Table S1); kinetic models and parameter constraints (Table S2); Arrhenius-based modeling equations (Table S3); smoothing filter performance comparisons (Tables S4–S5); supplementary kinetic model fitting results (Table S6) (PDF)
