# Supplementary material for: Pressure Sensing and Kinetic Modeling of Oxygen-Releasing Endoperoxides for Chemically Driven Micro-Actuation
Source: ACS Omega. 2025 Sep 24;10(39):46071–8. doi: 10.1021/acsomega.5c07255 (PMC12508970; doi:10.1021/acsomega.5c07255)
Supplement: Supplementary file 1 [file ao5c07255_si_001.pdf]

Supporting Information

Pressure Sensing and Kinetic Modeling of Oxygen-Releasing Endoperoxides for Chemically Driven Micro-Actuation

Abhishek Sharma[1,2, *], Vanessa Barth[1,3], Henning J. Jessen[1,3], Laura M. Comella[1,4]

[1] Cluster of Excellence livMatS @ FIT-Freiburg Center for Interactive Materials and Bioinspired Technologies, University of Freiburg, 79110 Freiburg, Germany

[2] Laboratory for the Design of Microsystems, Department of Microsystems Engineering (IMTEK), University of Freiburg, 79110 Freiburg, Germany

[3] Institute of Organic Chemistry, University of Freiburg, 79104 Freiburg, Germany

[4] Institute of Energy Efficient Mobility, Karlsruhe University of Applied Sciences, 76133 Karlsruhe, Germany

*Corresponding author: abhishek.sharma@livmats.uni-freiburg.de

1.1 *Chemical Synthesis Pathway of Anthracene-Endoperoxide (ANT-EPO)*

General Remarks:

Reagents were purchased from commercial suppliers and used without further purification. Moisture- or air-sensitive reactions were carried out under dry nitrogen or argon atmosphere in oven dried glassware, which is also called inert conditions.

Solvents were purchased in analytical grade and used without further purification. Anhydrous solvents were purchased from commercially suppliers (ACROS, SIGMA ALDRICH).

Thin layer chromatography was carried out on MERCK silica gel 60 F_{254} plates (0.25 mm layer thickness, fluorescence indicator) and visualized by UV light (λ = 254, 365 nm).

Normal phase medium pressure liquid chromatography (NP-MPLC) was performed using *PuriFlash*®430 from INTERCHIM. PuriFlash® Silica HP Flash Column were purchased from INTERCHIM.

Nuclear magnetic resonance (NMR) spectra were measured on a *Bruker Avance II* (^{1}H = 400 MHz) NMR spectrometer. The spectra were analyzed with the software *MestreNova* from MESTRELAB RESEARCH and signals were referenced to the internal solvent signal (^{1}H-NMR: $CDCl_3$ δ = 7.26 ppm, DMSO-d_6 δ = 2.50 ppm; ^{13}C-NMR: $CDCl_3$ δ = 77.16 ppm, DMSO-d_6 δ = 39.52). ^{19}F-NMR spectra were referenced to an external standard. Chemical shifts are quoted in ppm and coupling constants *J* were given in Hertz. The common abbreviations were used to characterize the signal multiplicity (singlet (s), doublet (d), triplet (t), quartet (q), multiplet (m), centered multiplet (m_c)).

High resolution mass spectra (HRMS) were measured by C. Warth and T. Huck (Analytical department of the university of Freiburg, institute of organic chemistry) on a *Thermo LCQ Advantage* (spray voltage: 2.5 – 4.5 kV, spray current: 5 µA, ion transfer tube: 250 (150) °C, evaporation temperature: 50 – 400 °C).

Synthesis procedures

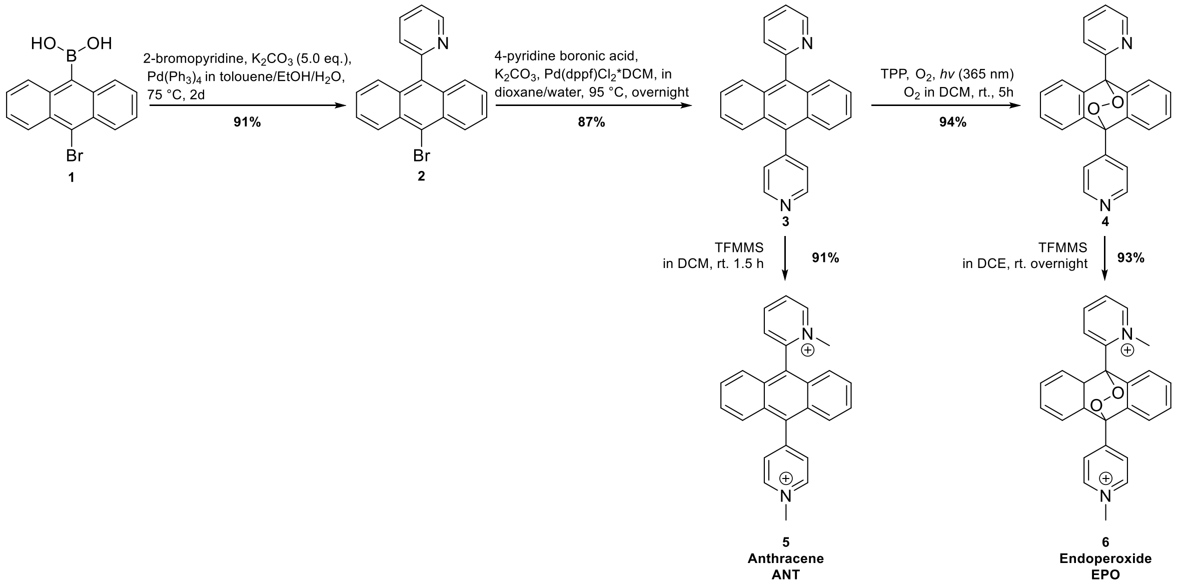

Figure S1. Overview of the reaction pathway to anthracene **5** *and endoperoxide* **6***, based on X. WANG.*[1]

Synthesis of 2-(10-bromoanthracen-9-yl)pyridine 2

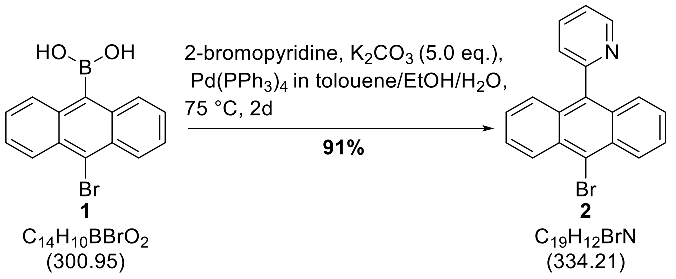

Figure S2. Reaction scheme for synthesis of 2-(10-bromoanthracen-9-yl)pyridine 2.

The reaction was performed under inert conditions.

A solution of (10-bromoantracen.9-yl) boronic acid **1** (1.00 g, 3.32 mmol) and 2-bromopyridine (412 µl, 683 mg, 4.32 mmol, 1.3 eq.) in toluene/ethanol/H_2O (1:1:1, 60 ml) was degassed under argon for 15 min. Afterwards, $Pd(PPh_3)_4$ (192 mg, 0.166 mmol, 5 mol-%) and K_2CO_3 (2.29 g, 16.6 mmol, 5.0 eq.) was added and the reaction mixture was stirred at 75 °C for 2 days. After cooling to room temperature, the reaction mixture was extracted with EA (3×) and the combined organic layers were dried over Na_2SO_4. The solvent was removed under reduced pressure and the residue was purified by NP-MPLC (30 µm SiHP, increasing concentration of EA in DCM, compound eluted at 2% EA, monitored at 254 nm) to obtain the title compound **2** (1.01 g, 3.02 mmol, 91%) as a yellow solid.

The title compound was prepared following a modified literature procedure.[2]

$\boldsymbol{R}_f$ (SiO_2, DCM/EA 50:1 (vol/vol)) 0.56.

^{1}H-NMR (400 MHz, $CDCl_3$, δ/ppm): 8.92 (ddd, J = 4.9, 1.9, 1.0 Hz, 1H), 8.63 (ddd, J = 8.9, 1.0 Hz, 2H), 7.93 (ddd, J = 7.7, 1.8 Hz, 1H), 7.59 (ddd, J = 8.9, 6.5, 1.2 Hz, 2H), 7.56 – 7.46 (m, 4H), 7.40 (ddd, J = 8.8, 6.5, 1.2 Hz, 2H).

^{13}C{^{1}H}-NMR (101 MHz, $CDCl_3$, δ/ppm): 158.0, 150.2, 136.4, 135.9, 130.9, 130.4, 128.1, 127.0, 126.9, 126.6, 126.2, 123.9, 122.7.

HRMS(ESI+): m/z calculated for $C_{19}H_{13}NBr^+$ $[M+H]^+$ 334.0226, found 334.0229.

Synthesis of 2-(10-(pyridin-4-yl)anthracen-9-yl)pyridine 3

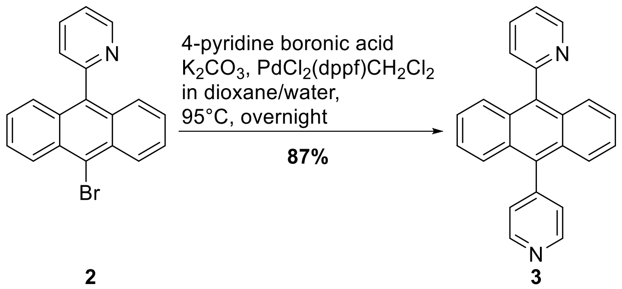

2
$C_{19}H_{12}BrN$
(334.22)

3
$C_{24}H_{16}N_2$
(332.41)

Figure S3. Reaction scheme for synthesis of 2-(10-(pyridin-4-yl)anthracen-9-yl)pyridine 3.

The reaction was performed under inert conditions.

A solution of **2** (393 mg, 1.18 mmol), 4-pyridine boronic acid (188 mg, 1.53 mmol, 1.3 eq.) and K_2CO_3 (818 mg, 5.88 mmol, 5.0 eq.) in dioxane/H_2O (2.5:1, 11 ml) was degassed for 20 min. Afterwards, Pd(dppf)Cl_2*DCM (48.0 mg, 58.7 µmol, 5 mol-%) was added and the reaction mixture was stirred at 95 °C overnight. After cooling to room temperature, the reaction mixture was extracted with EA (3×), the combined organic layers were washed with brine and dried over Na_2SO_4. The solvent was removed under reduced pressure and the residue was purified by NP-MPLC (30 µm SiHP, increasing concentration of EA in DCM, compound eluted at 40% EA, monitored at 254 nm) to obtain the title compound **3** (339 mg, 1.02 mmol, 87%) as a yellow solid.

The title compound was prepared following a modified literature procedure and analytical data were consistent with those found in literature.[1]

***R*f** (SiO_2, DCM/EA 2:1 (vol/vol)) 0.16.

^{1}H-NMR (400 MHz, $CDCl_3$, δ/ppm): 8.94 (ddd, *J* = 4.9, 1.9, 1.0 Hz, 1H), 8.90 – 8.85 (m, 2H), 7.96 (ddd, *J* = 7.7, 1.8 Hz, 1H), 7.63 – 7.56 (m, 5H), 7.52 – 7.40 (m, 3H), 7.40 – 7.33 (m, 4H).

^{13}C{^{1}H}-NMR (101 MHz, $CDCl_3$, δ/ppm): 158.3, 150.3, 150.1, 150.0, 147.8, 136.5, 136.3, 134.7, 129.7, 129.3, 126.8, 126.8, 126.5, 126.5, 126.2, 125.8, 122.6.

HRMS(ESI+): m/z calculated for $C_{24}H_{17}N_2^+$ [M+H] 333.1386, found 333.1389.

***Synthesis of 2-(10-(pyridin-4-yl)-9,10-epidioxyanthracen-9(10H)-yl)pyridine* 4**

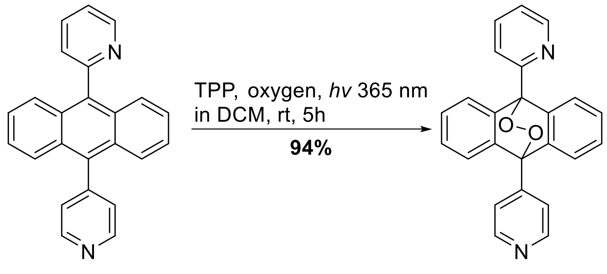

3
$C_{24}H_{16}N_2$
(332.41)

4
$C_{24}H_{16}N_2O_2$
(364.40)

Figure S4. Reaction scheme for synthesis of 2-(10-(pyridin-4-yl)-9,10-epidioxyanthracen-9(10H)-yl)pyridine 4.

3 (160 mg, 481 µmol) and tetraphenylporphyrin, (TPP, 3.90 mg, 6.34 µmol, 1.3 mol-%) were dissolved in DCM (15 ml) and oxygen was bubbled through the solution while stirring for 15 min. The mixture was kept under an oxygen atmosphere with a balloon and irradiated for 5 h with a 365 nm-LED (Mightex, LCS-0365-04, 48 mm aperture, 500 mW). The solvent was removed under reduced pressure and the residue was purified by NP-MPLC (30 µm SiHP, increasing concentration of EA in DCM, compound eluted at 47% EA, monitored at 254 nm) to obtain the title compound **4** (164 mg, 450 µmol, 94%) as a white solid.

The title compound was prepared following a modified literature procedure and analytical data were consistent with those found in literature.[1]

***R*f** (SiO_2, DCM/EA 4:1 (vol/vol)) 0.52.

^{1}H-NMR (400 MHz, $CDCl_3$, δ/ppm): 8.97 (ddd, *J* = 4.8, 1.9, 1.0 Hz, 1H), 8.92 – 8.89 (m, 2H), 7.95 (ddd, *J* = 7.8, 1.8 Hz, 1H), 7.78 (ddd, *J* = 8.0, 1.1 Hz, 1H), 7.67 – 7.63 (m, 2H), 7.50 (ddd, *J* = 7.6, 4.8, 1.2 Hz, 1H), 7.25 – 7.19 (m, 6H), 7.10 – 7.05 (m, 2H).

^{13}C{^{1}H}-NMR (101 MHz, $CDCl_3$, δ/ppm): 153.8, 149.9, 148.9, 142.1, 140.1, 138.6, 137.6, 128.2, 127.8, 123.9, 123.6, 122.7, 122.6, 122.4, 84.3, 83.5.

HRMS(ESI+): m/z calculated for $C_{24}H_{17}O_2N_2^+$ $[M+H]^+$ 365.1285, found 365.1286.

***Synthesis of 1-methyl-2-(10-(1-methylpyridin-1-ium-4-yl)anthracen-9-yl)pyridin-1-ium* 5**

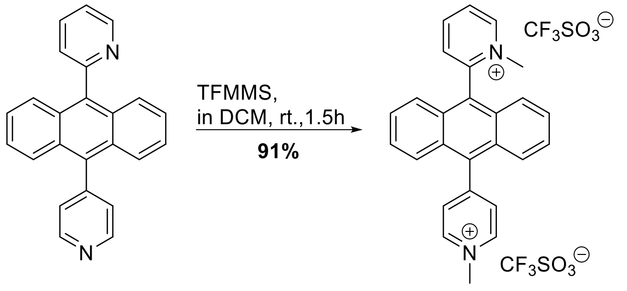

3
$C_{24}H_{16}N_2$
(332.41)

5
$C_{26}H_{22}N_2^{2+}$
(326.47)
with counterion:
(660.08)

Figure S5. Reaction scheme for synthesis of 1-methyl-2-(10-(1-methylpyridin-1-ium-4-yl)anthracen-9-yl)pyridin-1-ium 5.

To a solution of **3** (11.0 mg, 33.0 µmol) in dry DCM (2 ml) methyl triflouromethanesulfonate (TFMMS, 17.2 µL, 25.0 mg, 0.152 mmol, 4.6 eq.) was added at 0 °C. After 30 min, a precipitate was formed and the reaction mixture was allowed to warm to room temperature and stirred for further 1 h. The precipitate was collected by centrifugation and washed twice with DCM to obtain the title compound **5** (19.9 mg, 30.1 µmol, 91%) as a yellow solid.

The title compound was prepared following a literature procedure and analytical data were consistent with those found in literature.[1]

^{1}H-NMR (400 MHz, DMSO-d$_6$, δ/ppm): 9.45 (d, J = 6.1 Hz, 1H), 9.32 – 9.24 (m, 2H), 8.87 (ddd, J = 7.9, 1.1 Hz, 1H), 8.46 (ddd, J = 7.9, 6.2, 1.5 Hz, 1H), 8.37 – 8.32 (m, 2H), 8.29 (dd, J = 8.3, 1.3 Hz, 1H), 7.72 – 7.62 (m, 6H), 7.58 – 7.53 (m, 2H), 4.54 (s, 3H), 3.91 (s, 3H).

^{13}C{^{1}H}-NMR (101 MHz, DMSO-d$_6$, δ/ppm): 154.2, 151.7, 149.0, 146.2, 146.0, 134.9, 131.4, 129.8, 128.7, 128.5, 128.0, 128.0, 127.5, 126.3, 126.1, 124.8,120.7 (q, $^1J_{CF}$ = 322 MHz, triflate), 47.8, 46.0.

^{19}F-NMR (377 MHz, DMSO-d_6, δ/ppm): −77.72.

HRMS(ESI+): m/z calculated for $C_{26}H_{22}N_2^+$ $[M]^{2+}$ 181.0886, found 181.0885.

Synthesis of 1-methyl-2-(10-(1-methylpyridin-1-ium-4-yl)-9,10-epidioxyanthracen-9(10H)-yl)pyridin-1-ium **6**

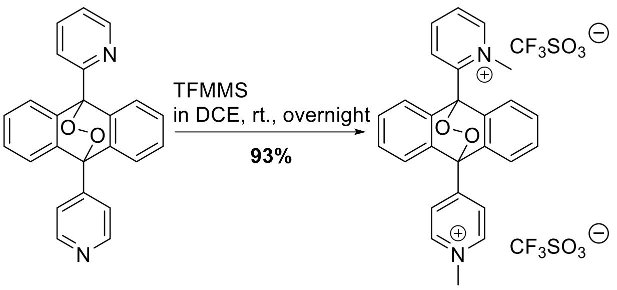

4
$C_{24}H_{16}N_2O_2$
(364.40)

6
$C_{26}H_{22}N_2O_2^{2+}$
(394.47)
with counterion
$C_{28}H_{22}F_6N_2O_8S_2$
(692.60)

Figure S6. Reaction scheme for synthesis of 1-methyl-2-(10-(1-methylpyridin-1-ium-4-yl)-9,10-epidioxyanthracen-9(10H)-yl)pyridin-1-ium 6.

To a solution of **4** (130 mg, 0.357 mmol) in dry 1,2-dichloroethane (DCE, 22 ml) methyl triflouromethanesulfonate (TFMMS, 404 µL, 585 mg, 3.57 mmol, 10.0 eq.) was added at 0 °C. The reaction mixture was allowed to warm to room temperature and stirred further overnight. The formed precipitate was collected by centrifugation and afterwards solved in DMSO (1.8 ml). This solution was then dripped into a premixed solution of n-pentane/DCM (20 ml, 1:1) to precipitate the product. The precipitate was washed twice with DCM to obtain the title compound **5** (230 mg, 0.332 mmol, 93%) as a white solid.*

The title compound was prepared following a modified literature procedure and analytical data were consistent with those found in literature.[1]

^{1}H-NMR (400 MHz, DMSO-d$_6$, δ/ppm): 9.45 (dd, J = 6.3, 1.5 Hz, 1H), 9.32 (d, J = 6.4 Hz, 2H), 8.90 (td, J = 8.0, 1.5 Hz, 1H), 8.54 – 8.45 (m, 3H), 8.40 (dd, J = 8.3, 1.5 Hz, 1H), 7.58 – 7.46 (m, 6H), 7.26 – 7.18 (m, 2H), 4.53 (s, 3H), 4.26 (s, 3H).

^{13}C{^{1}H}-NMR (101 MHz, DMSO-d$_6$, δ/ppm): 151.8, 148.0, 147.3, 146.9, 146.5, 136.0, 133.8, 129.7, 129.6, 128.3, 126.5, 126.0, 124.2, 123.5, 120.7 (q, $^1J_{CF}$ = 322 MHz, triflate), 84.3, 82.2, 49.7, 47.9.

^{19}F-NMR (377 MHz, DMSO-d_6, δ/ppm): −77.72.

HRMS (ESI+): m/z calculated for $C_{26}H_{22}O_2N_2^{2+}$ $[M]^{2+}$ 197.0835, found 197.0837.

* To obtain a very high purity, it can be necessary to repeat the solving in DMSO and subsequent precipitation in n-pentane/DCM. This reduces the monomethlylated by-product.

Supporting References:

(1) Wang, X.; Bittner, T.; Milanov, M.; Kaul, L.; Mundinger, S.; Koch, H.-G.; Jessen-Trefzer, C.; Jessen, H. J. Pyridinium Modified Anthracenes and Their Endoperoxides Provide a Tunable Scaffold with Activity against Gram-Positive and Gram-Negative Bacteria. *ACS infectious diseases* 2021, *7* (8), 2073–2080. DOI: 10.1021/acsinfecdis.1c00263. Published Online: Jul. 22, 2021.

(2) G. Haykir, E. Tekin, T. Atalar, F. Türksoy, *Thin Solid Films* 2013, *548*, 171.

NMR result

2-(10-bromoanthracen-9-yl)pyridine 2

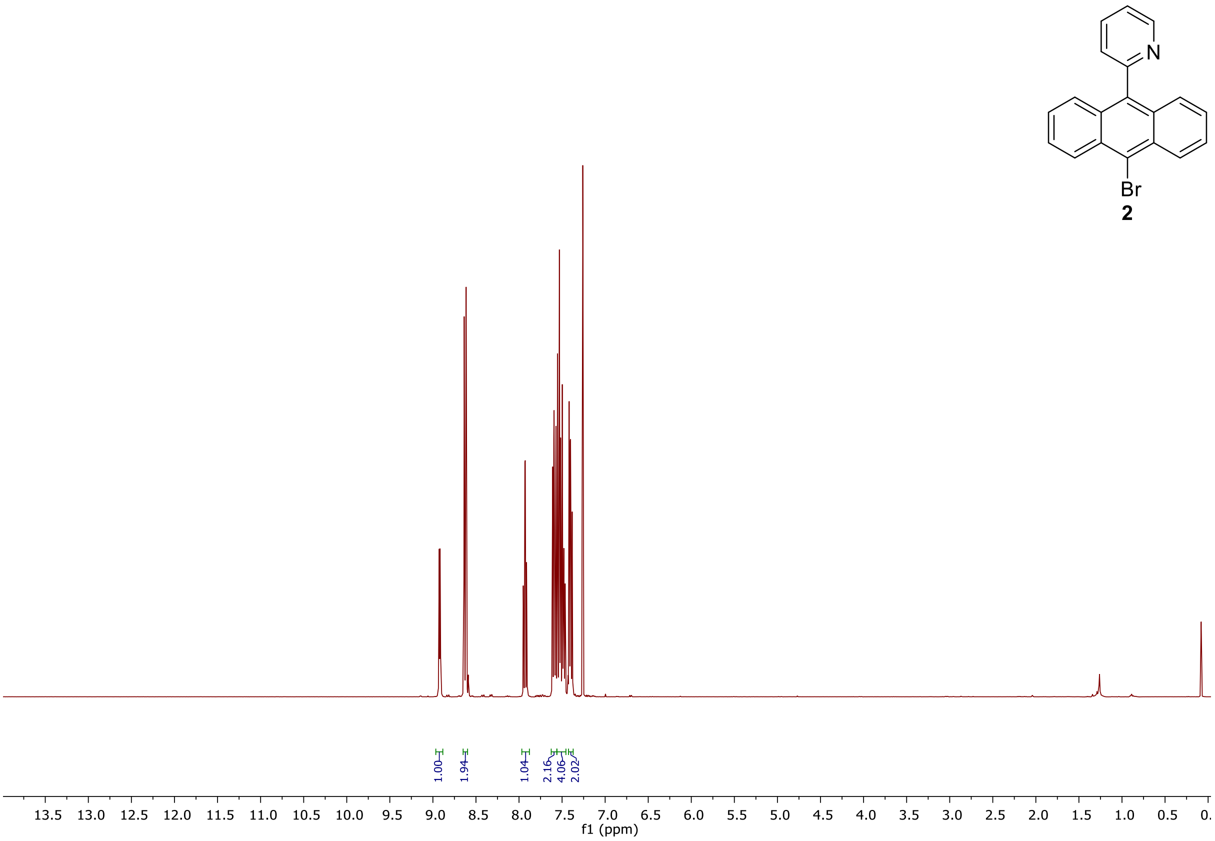

Figure S7. ^{1}*H-NMR-spectrum (400 MHz) of compound* ***2****, measured in* $CDCl_3$ *at 298 K.*

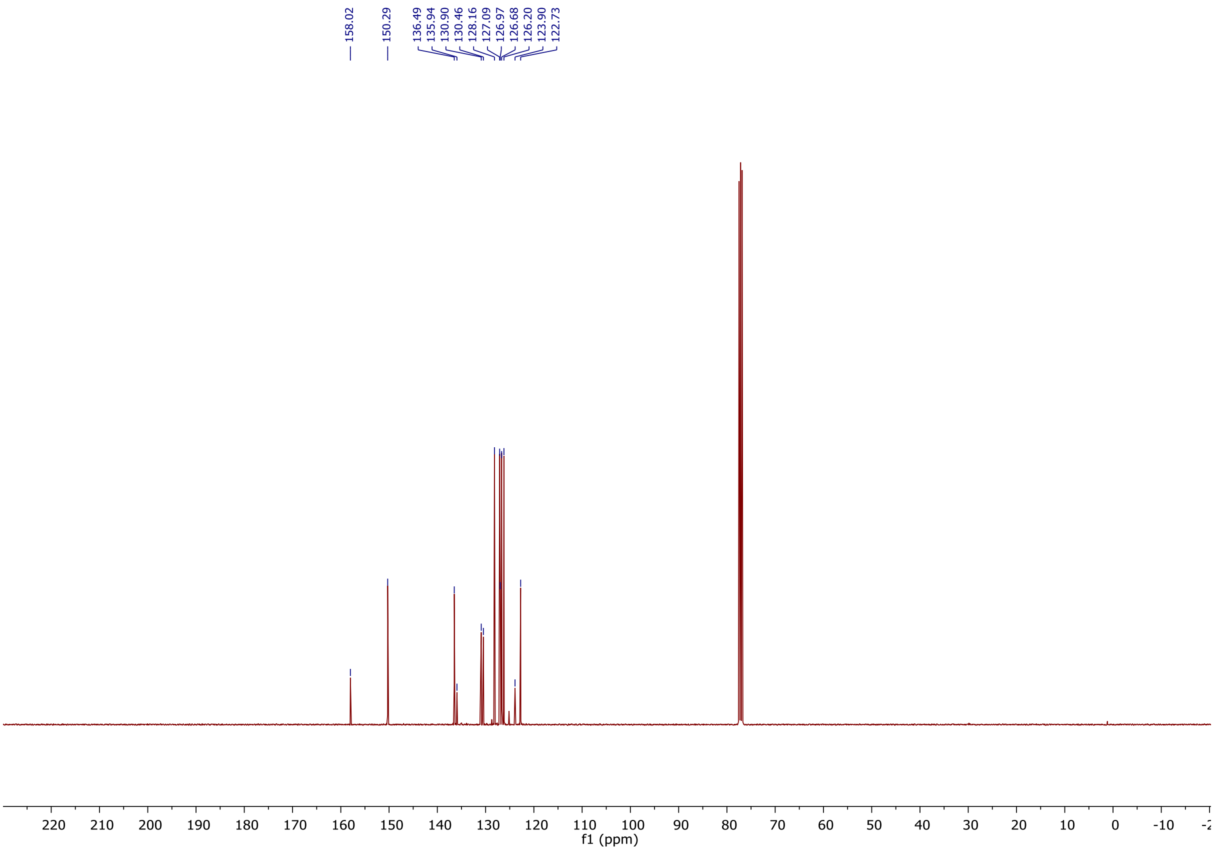

Figure S8. $^{13}C\{^{1}H\}$*-NMR-spectrum (101 MHz) of compound* ***2****, measured in* $CDCl_3$ *at 298 K.*

2-(10-(pyridin-4-yl)anthracen-9-yl)pyridine **3**

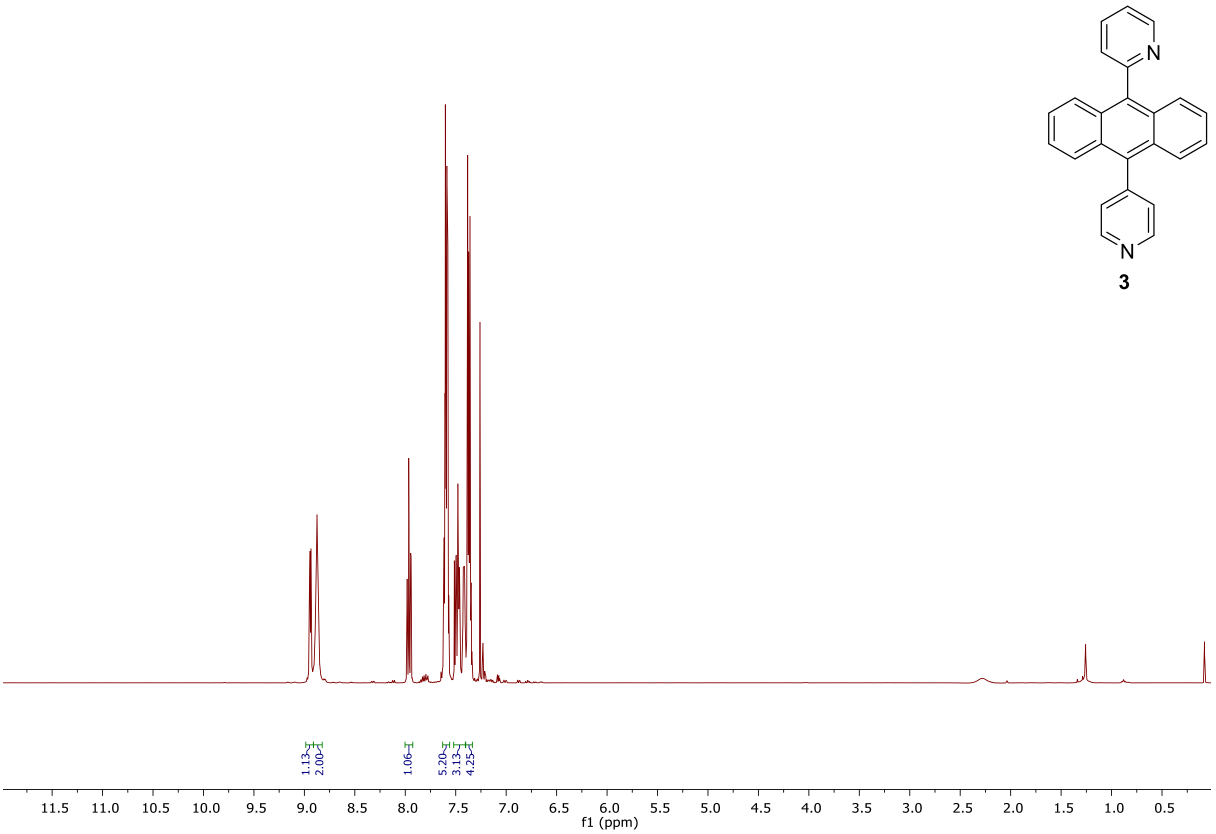

Figure S9. ^{1}H*-NMR-spectrum (400 MHz) of compound* ***3****, measured in* $CDCl_3$ *at 298 K.*

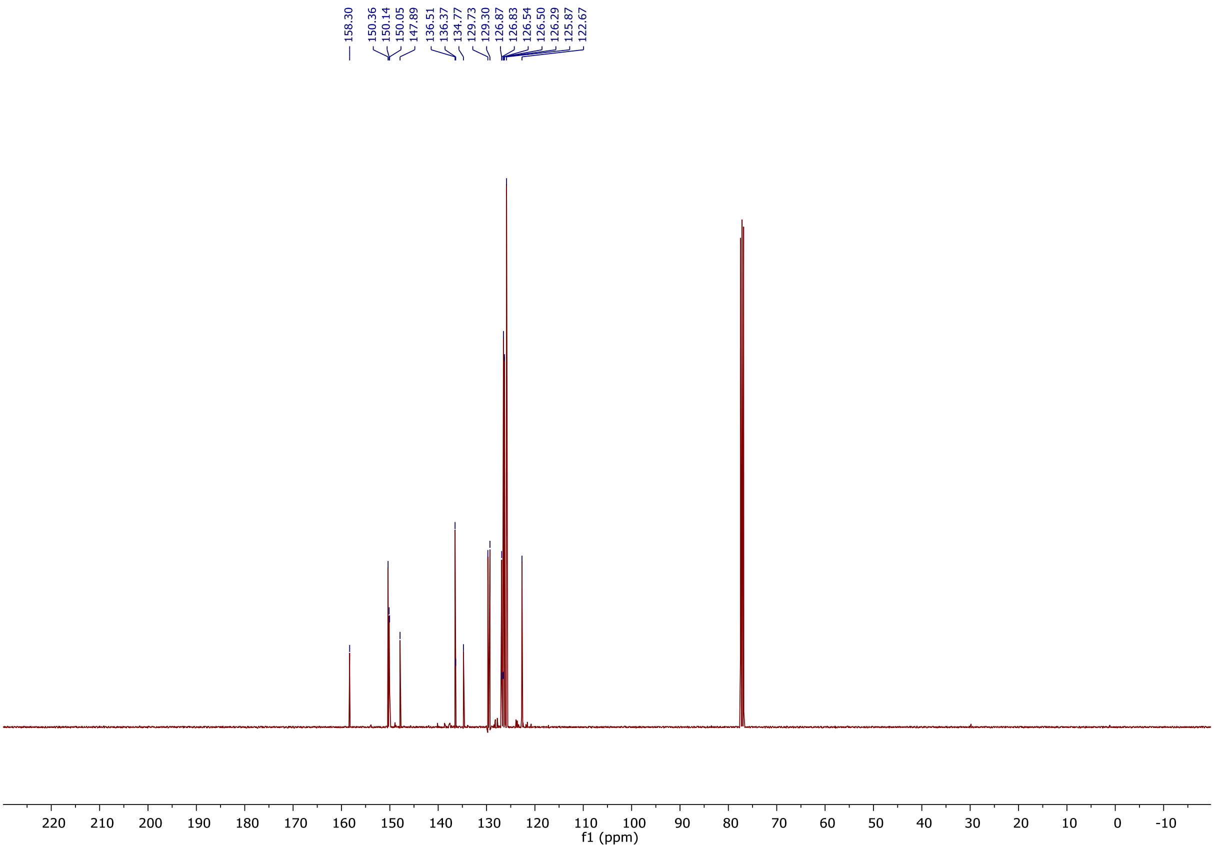

Figure S10. $^{13}C\{^{1}H\}$*-NMR-spectrum (101 MHz) of compound* **3**, *measured in* $CDCl_3$ *at 298 K.*

2-(10-(pyridin-4-yl)-9,10-epidioxyanthracen-9(10H)-yl)pyridine 4

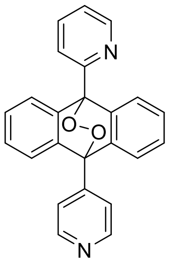

4

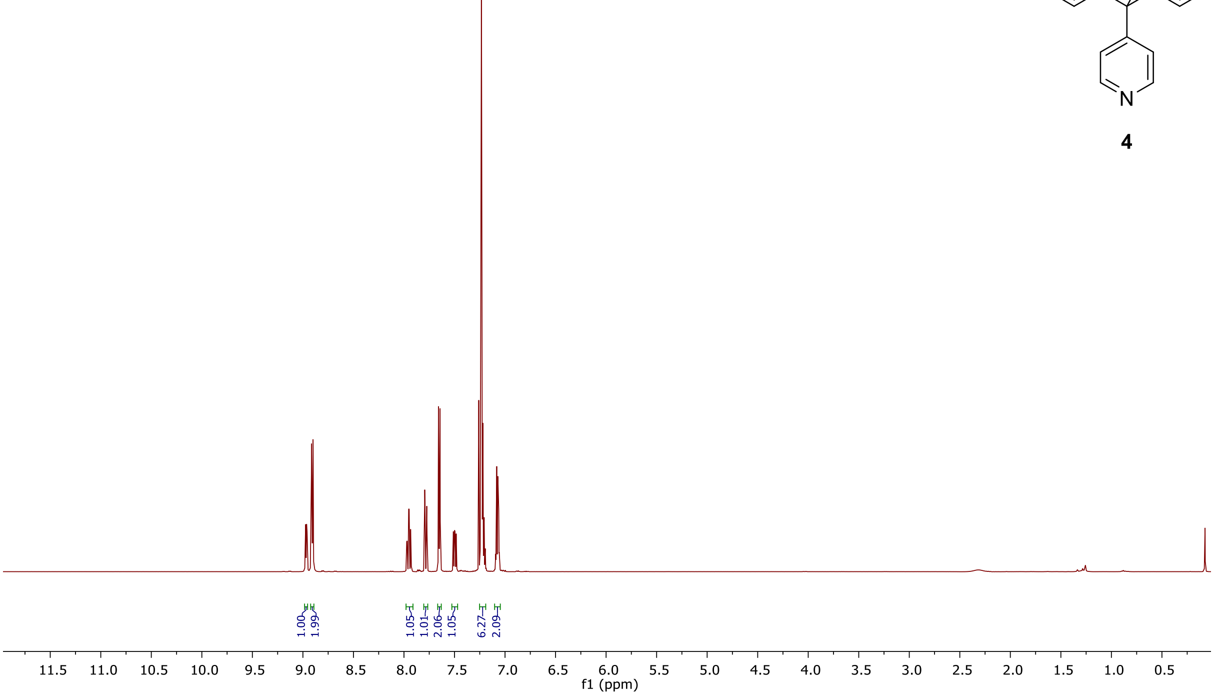

Figure S11. ^{1}H*-NMR-spectrum (400 MHz) of compound* **4**, *measured in* $CDCl_3$ *at 298 K.*

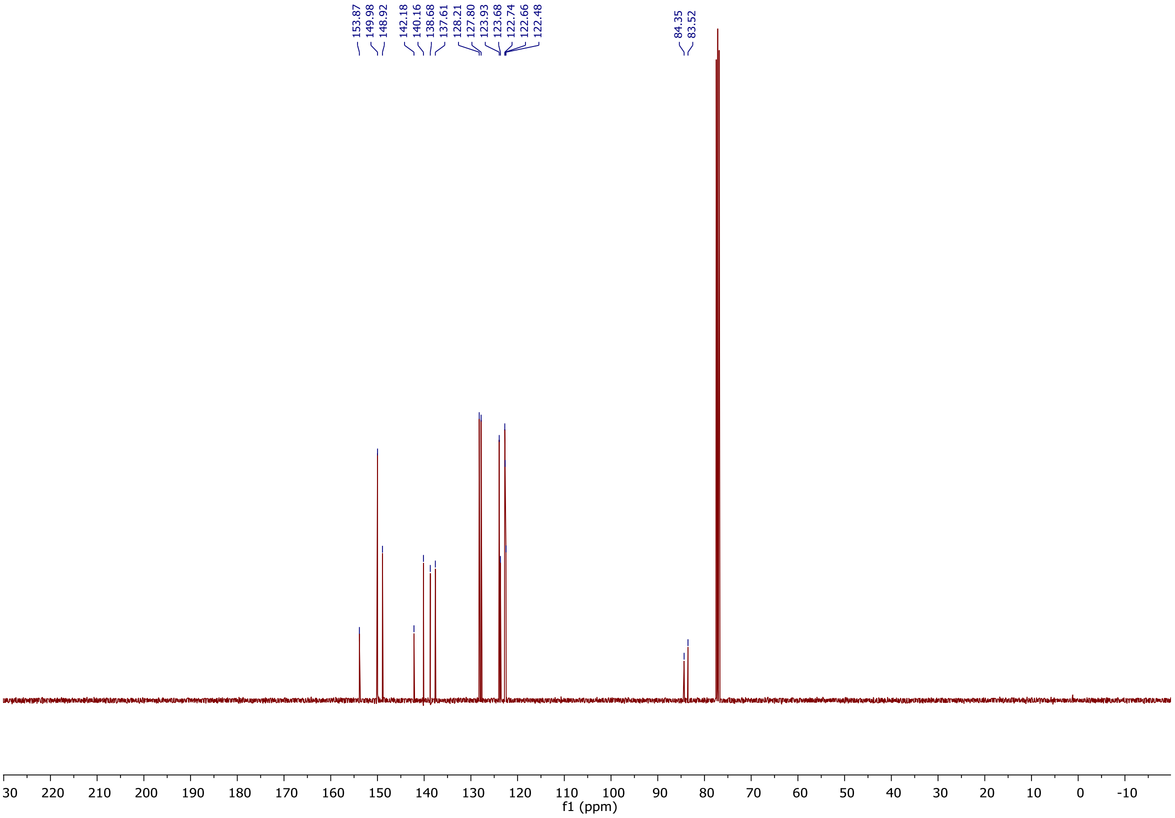

Figure S12. $^{13}C\{^{1}H\}$-NMR-spectrum (101 MHz) of compound **4***, measured in $CDCl_3$ at 298 K.*

1-methyl-2-(10-(1-methylpyridin-1-ium-4-yl)anthracen-9-yl)pyridin-1-ium 5

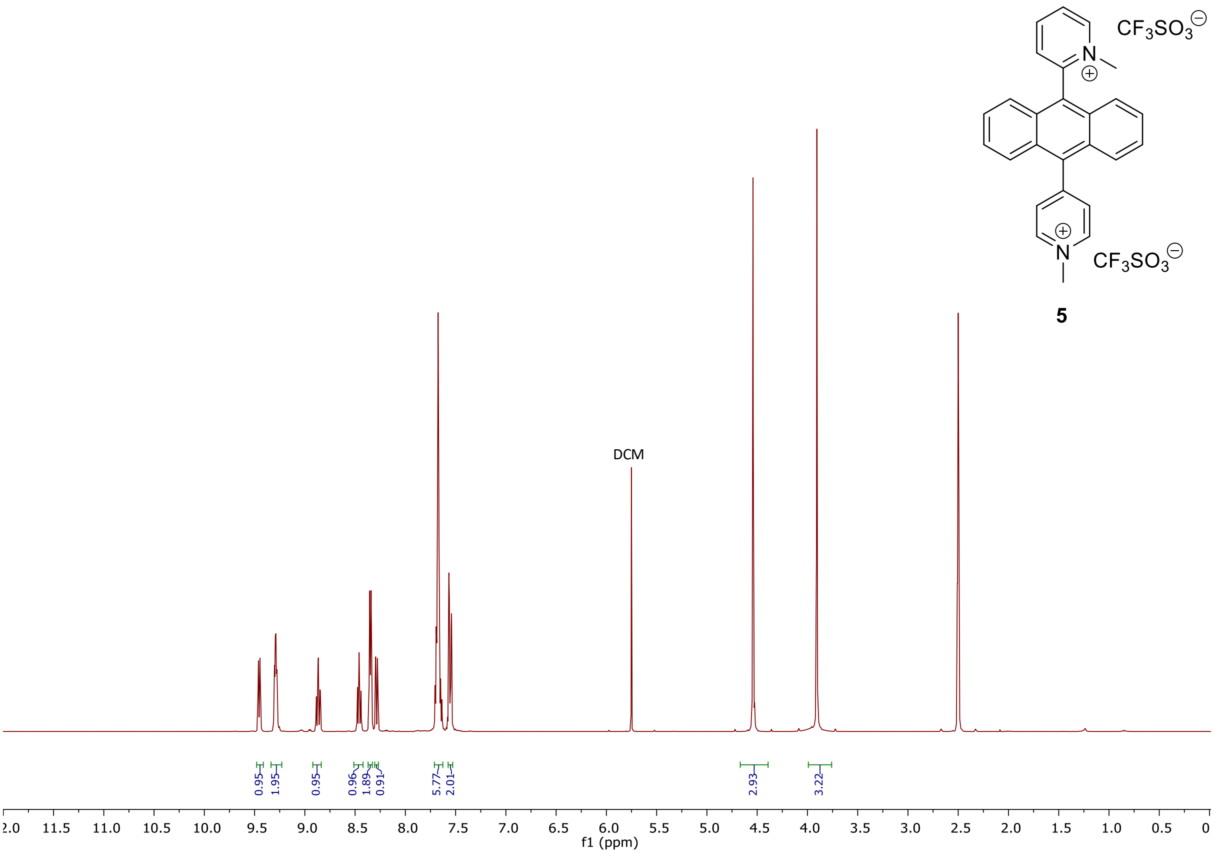

Figure S13. ^{1}H-NMR-spectrum (400 MHz) of compound **5***, measured in DMSO-d_6 at 298 K.*

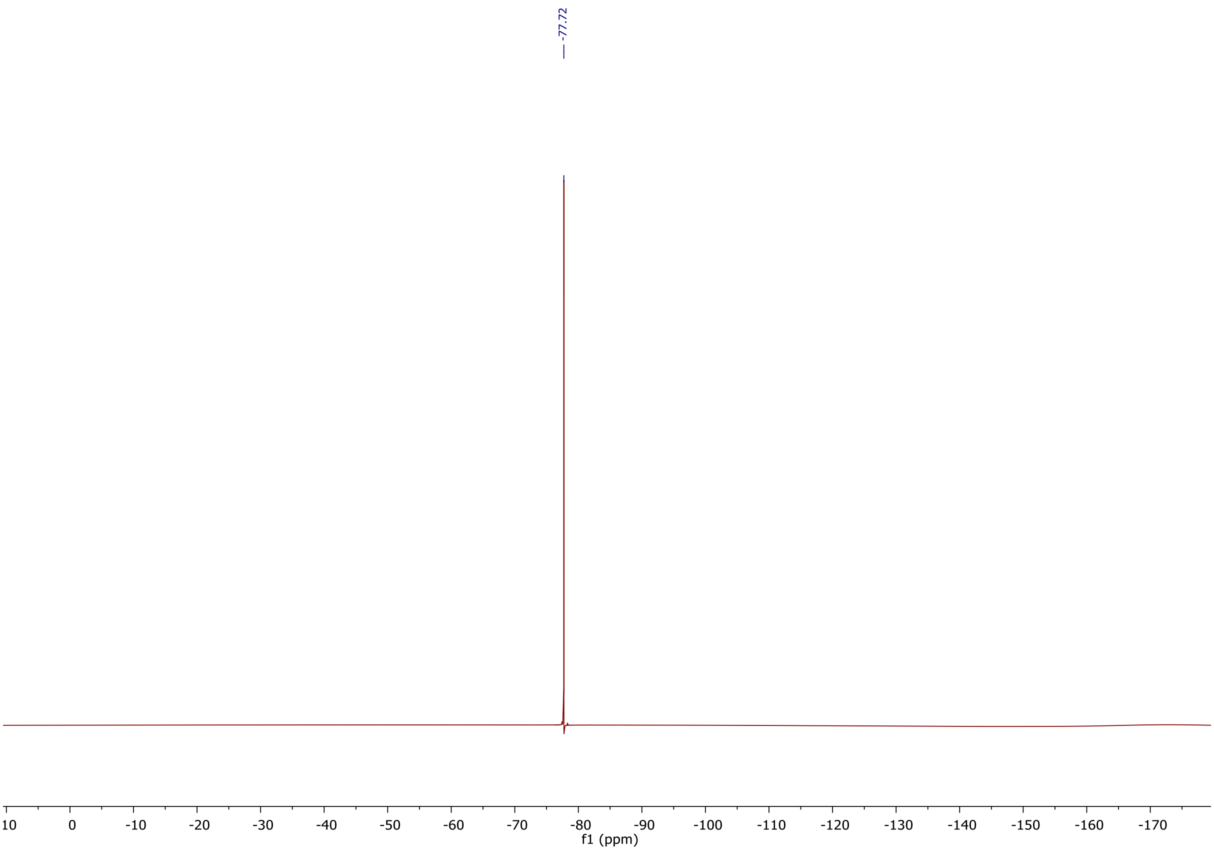

Figure S14. ^{19}*F-NMR-spectrum (377 MHz) of compound* **5***, measured in DMSO-d*$_6$ *at 298 K.*

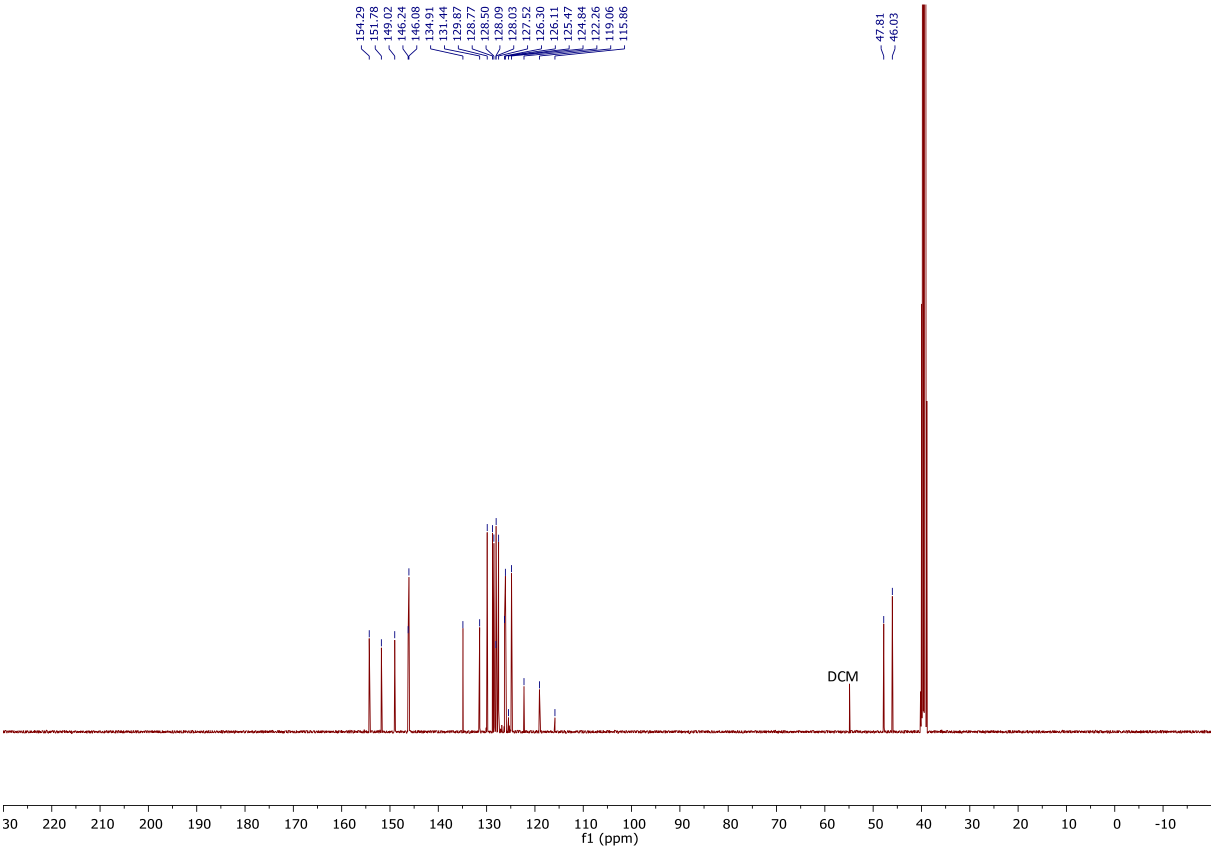

Figure S15. $^{13}C\{^1H\}$*-NMR-spectrum (101 MHz) of compound* **5***, measured in DMSO-d*$_6$ *at 298 K.*

***1-methyl-2-(10-(1-methylpyridin-1-ium-4-yl)-9,10-epidioxyanthracen-9(10H)-yl)pyridin-1-ium* 6**

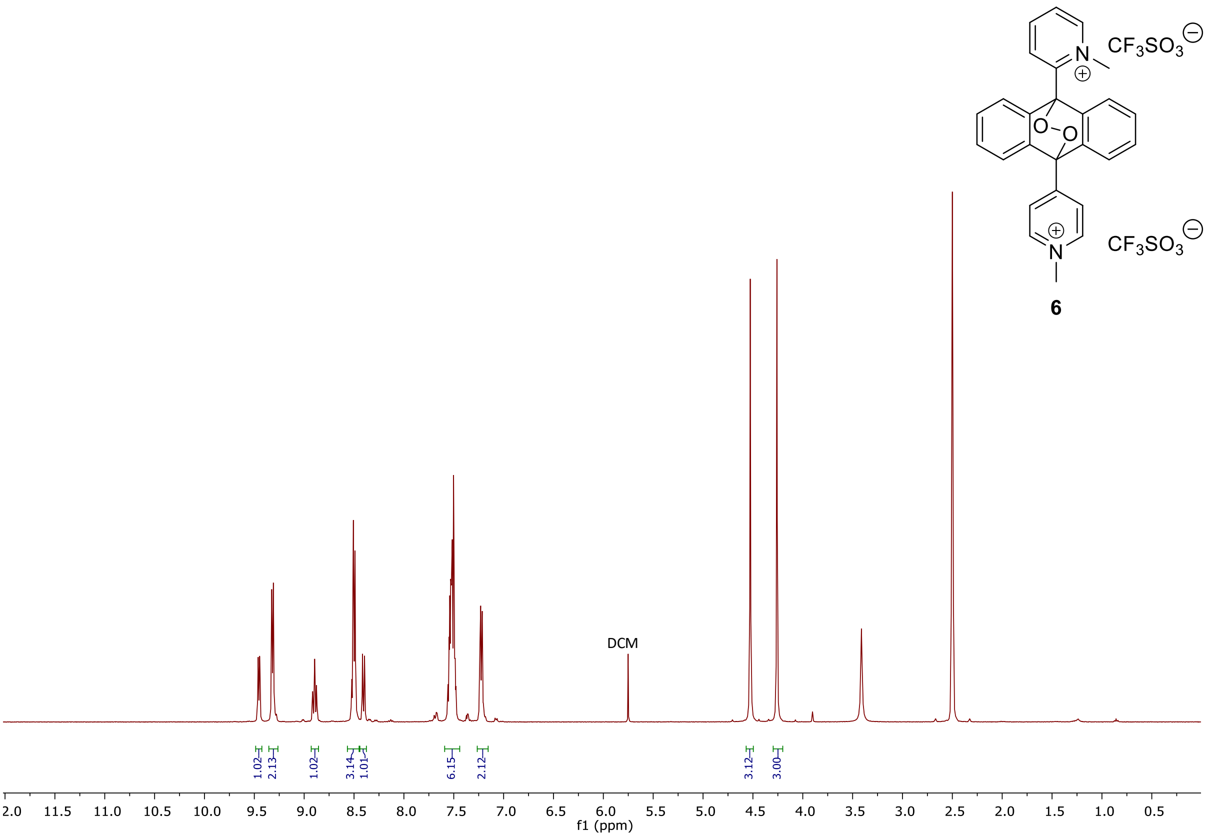

Figure S16. ^{1}H-NMR-spectrum (400 MHz) of compound **6***, measured in DMSO-d$_6$ at 298 K.*

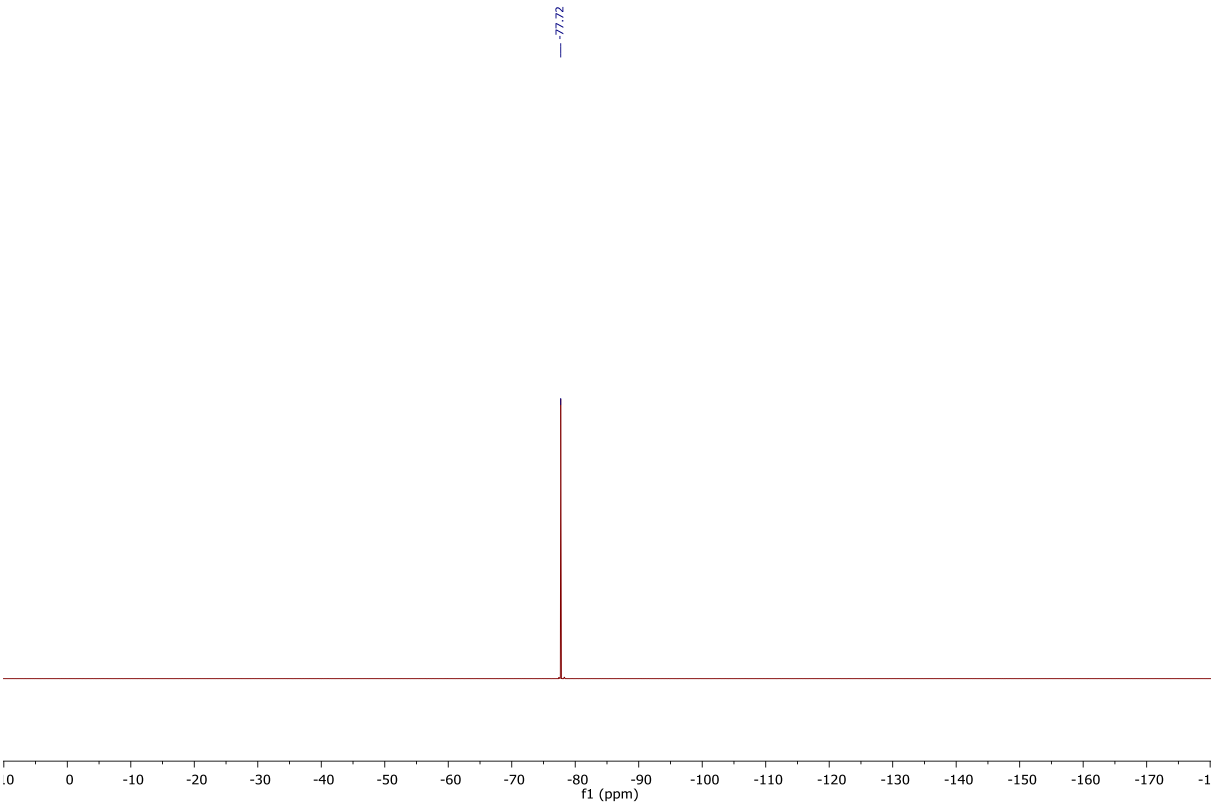

Figure S17. ^{19}F-NMR-spectrum (377 MHz) of compound **6,** *measured in DMSO-d$_6$ at 298 K.*

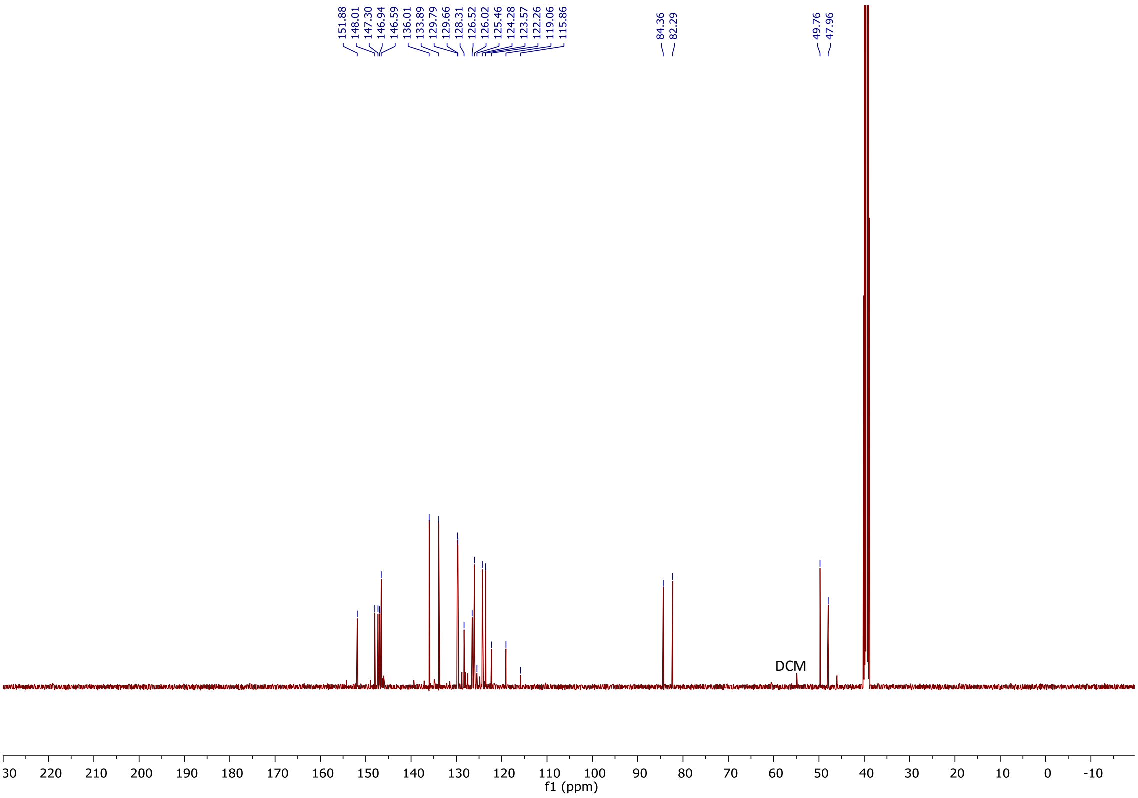

Figure S18. $^{13}C\{^1H\}$*-NMR-spectrum (101 MHz) of compound* **6**, *measured in DMSO-d_6 at 298 K.*

1.2 Measurement Analysis

Table S1. Adaptive Savitzky-Golay (SG) filter scoring function equation and parameter weights based on signal-to-noise ratio (SNR) and root-mean-square error (RMSE) optimization.

Metric	Description	Formula	Value/range
SNR	SNR improvement after filtering	$SNR = 10\log_{10}\left(\frac{Var(signal)}{Var(noise)}\right)$	Computed per window setting
RMSE	RMSE between raw and filtered signal	$RMSE = \sqrt{\frac{1}{N}\sum_{i=1}^{N}\left(P_i^{raw} - P_i^{filtered}\right)^2}$	
Composite score (S)	Weighted score to select optimal SG window	$Score = \alpha.SNR + \beta.RMSE$	α, β ϵ [0,1]
Window range	SG window sizes evaluated	Odd integers [9, 251]	Adaptive to signal duration

Table S2. Kinetic models used for fitting pressure-time data and respective boundary constraints.

Models	Computation	Boundary constraints
First order exponential	$P(t) = P_{max}\left(1 - e^{-k.t}\right)$	P_{max}: [0.8 × P_{max}, 1.2 × P_{max}];<br>k > 0
Logistic growth	$P(t) = \frac{P_{max}}{1 + exp^{-k(t-t_0)}}$	t_0: [0.8 × t_0, 1.2 × t_0];<br>k: [0, 1];<br>P_{max}: [0.8 × P_{max}, 1.2 × P_{max}]

* Where, P_{max} is the maximum pressure, k is the rate constant, and t_0 is the inflection point.

Table S3. Arrhenius-based temperature dependent kinetic modeling equations with fit boundaries.

Models	Computation	Boundary constraints
First-order (Arrhenius-based)	$P(t,T) = P_{max}\left(1 - e^{-k(T).t}\right).$	P_{max} > 0; $k(T)$ > 0
Logistic-growth (temperature dependent)	$P(t,T) = \frac{P_{max}}{1 + exp^{-k(T).(t-t_0)}}$	P_{max} > 0; $k(T)$ > 0; $t_0 \geq 0$
Rate constant, k (Arrhenius-based)	$k(T) = k_0 \, . exp^{\frac{-E_a}{RT}}$	k_0 > 0; E_a > 0;<br>R = 8.314 J/mol.K

* Where, T is temperature (k), R is the ideal gas constant, and E_a is the activation energy.

Table S4. Sample M_1, quantitative comparison of Savitzky–Golay (SG), moving average, and Gaussian smoothing filters applied to baseline-corrected pressure traces. Metrics include signal-to-noise ratio (SNR) and root-mean-square error (RMSE). SG filtering consistently achieves higher SNR and lower RMSE while preserving kinetic transitions.

Windows Size	Savitzky-Golay		Moving average		Gaussian smoothing	
	SNR (dB)	RMSE (kPa)	SNR (dB)	RMSE (kPa)	SNR (dB)	RMSE (kPa)
11	1.15517	0.00045	1.14379	0.00556	1.14392	0.00057
13	1.15550	0.00051	1.14366	0.00605	1.14381	0.00063
19	1.15738	0.00065	1.14336	0.00732	1.14352	0.00074
25	1.15779	0.00075	1.14315	0.00839	1.14332	0.00081
31	1.15798	0.00082	1.14310	0.00934	1.14318	0.00086
41	1.15822	0.00087	1.14291	0.01074	1.14301	0.00092
51	1.15845	0.00090	1.14282	0.01197	1.14290	0.00098
61	1.15855	0.00094	1.14270	0.01309	1.14279	0.00103
71	1.15863	0.00097	1.14254	0.01412	1.14270	0.00108

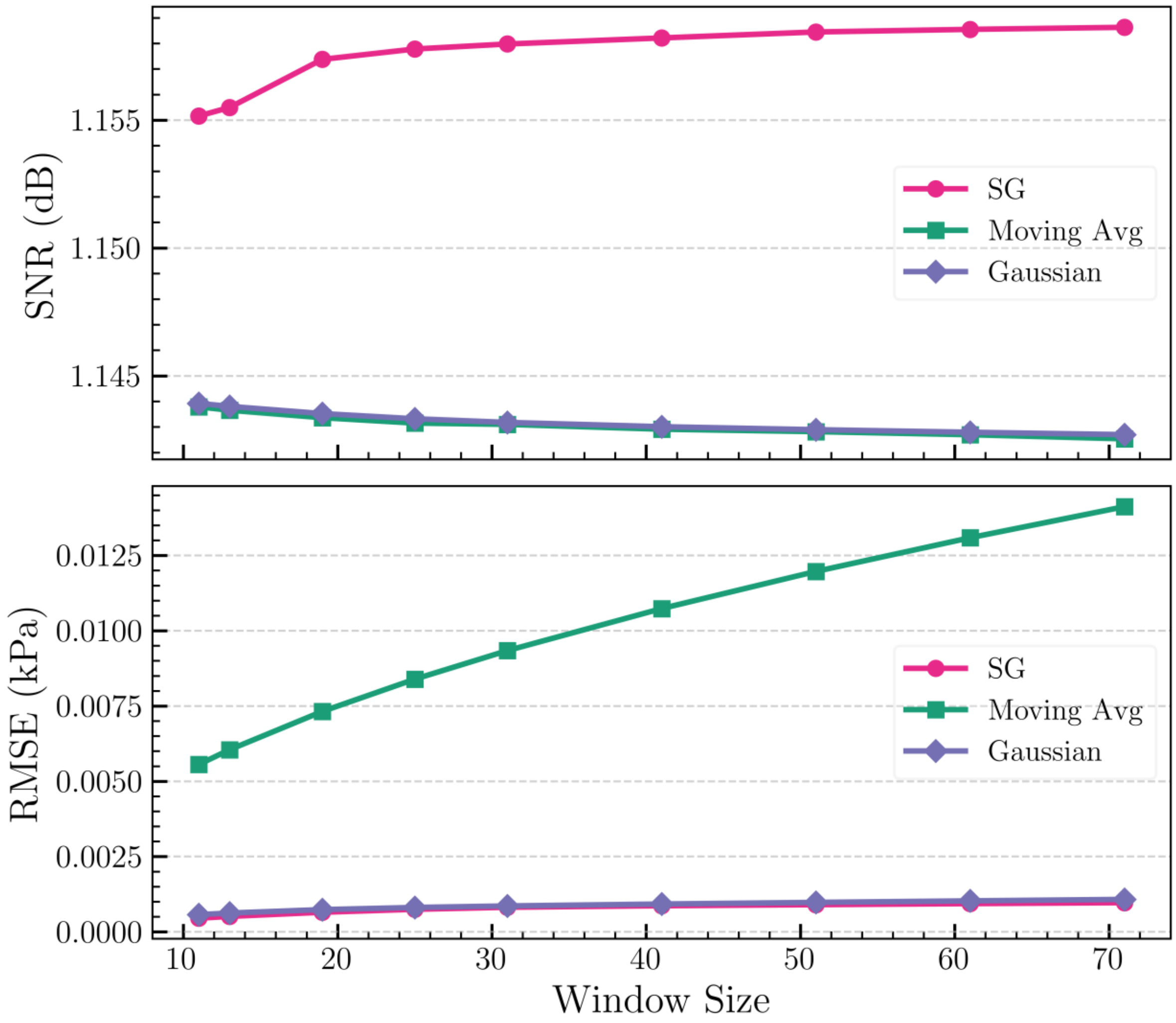

Figure S19. Sample M_1, comparison of smoothing performance across different window sizes for Savitzky–Golay (SG), moving average, and Gaussian filters. The top panel shows signal-to-noise ratio (SNR) improvement, while the bottom panel shows root-mean-square error (RMSE) relative to the raw baseline-corrected pressure signal. SG consistently achieves higher SNR and lower RMSE, especially in the 9–21 window range, supporting its selection for preserving signal fidelity during kinetic modeling.

Table S5. Sample M_2, quantitative comparison of Savitzky–Golay (SG), moving average, and Gaussian smoothing filters applied to baseline-corrected pressure traces. Metrics include signal-to-noise ratio (SNR) and root-mean-square error (RMSE). SG filtering consistently achieves higher SNR and lower RMSE while preserving kinetic transitions.

Windows Size	Savitzky-Golay		Moving average		Gaussian smoothing	
	SNR (dB)	RMSE (kPa)	SNR (dB)	RMSE (kPa)	SNR (dB)	RMSE (kPa)
11	0.87284	0.00054	0.87150	0.00454	0.87165	0.00072
13	0.87254	0.00062	0.87134	0.00494	0.87138	0.00079
19	0.87184	0.00082	0.87081	0.00598	0.87088	0.00095
25	0.87169	0.00096	0.87056	0.00685	0.87054	0.00106
31	0.87118	0.00105	0.87036	0.00762	0.87031	0.00114
41	0.87080	0.00115	0.87015	0.00875	0.87004	0.00123
51	0.87067	0.00122	0.86982	0.00974	0.86983	0.00131
61	0.87062	0.00128	0.86947	0.01064	0.86960	0.00137
71	0.87060	0.00133	0.86921	0.01147	0.86935	0.00142

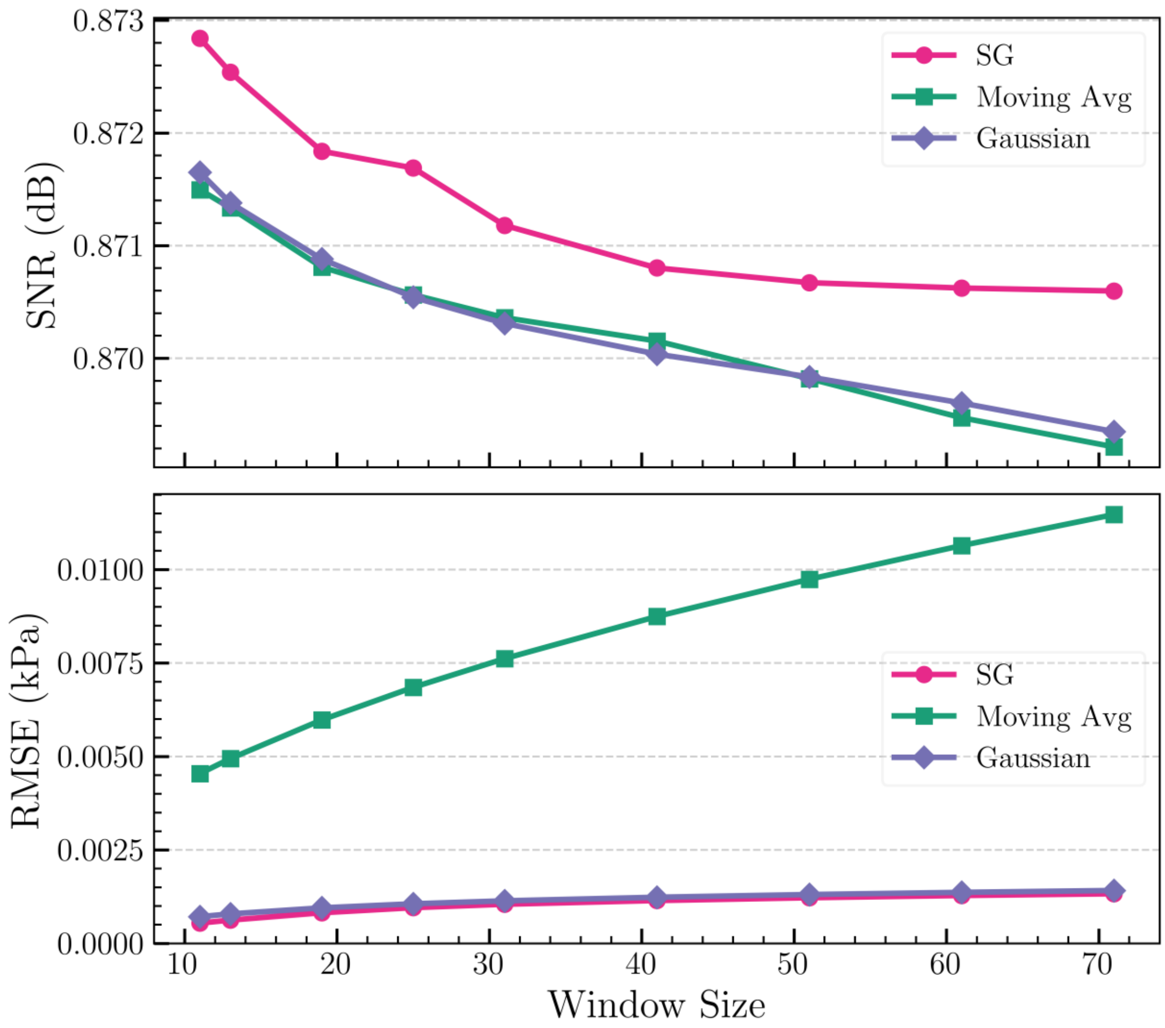

Figure S20. Sample M_2, comparison of smoothing performance across different window sizes for Savitzky–Golay (SG), moving average, and Gaussian filters. The top panel shows signal-to-noise ratio (SNR) improvement, while the bottom panel shows root-mean-square error (RMSE) relative to the raw baseline-corrected pressure signal. SG consistently achieves higher SNR and lower RMSE, especially in the 11–21 window range, supporting its selection for preserving signal fidelity during kinetic modeling.

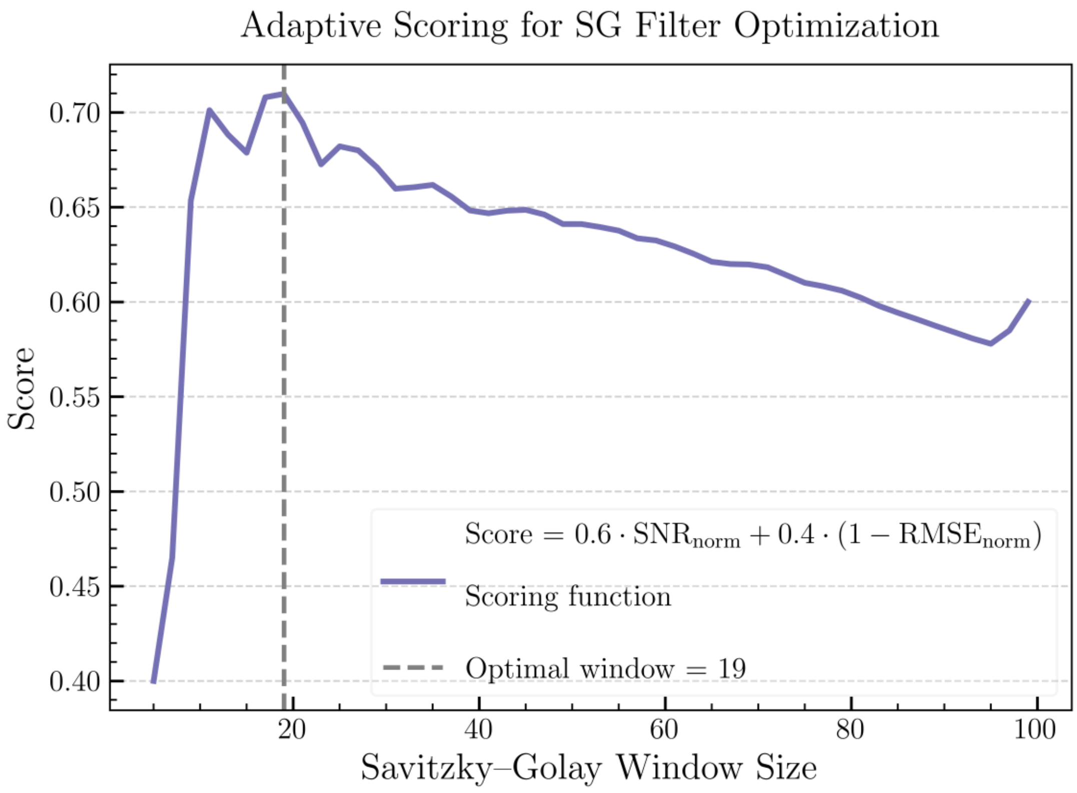

Figure S21. Effect of SG filter window size on SNR and RMSE for Sample M_1, used to determine the optimal adaptive smoothing configuration.

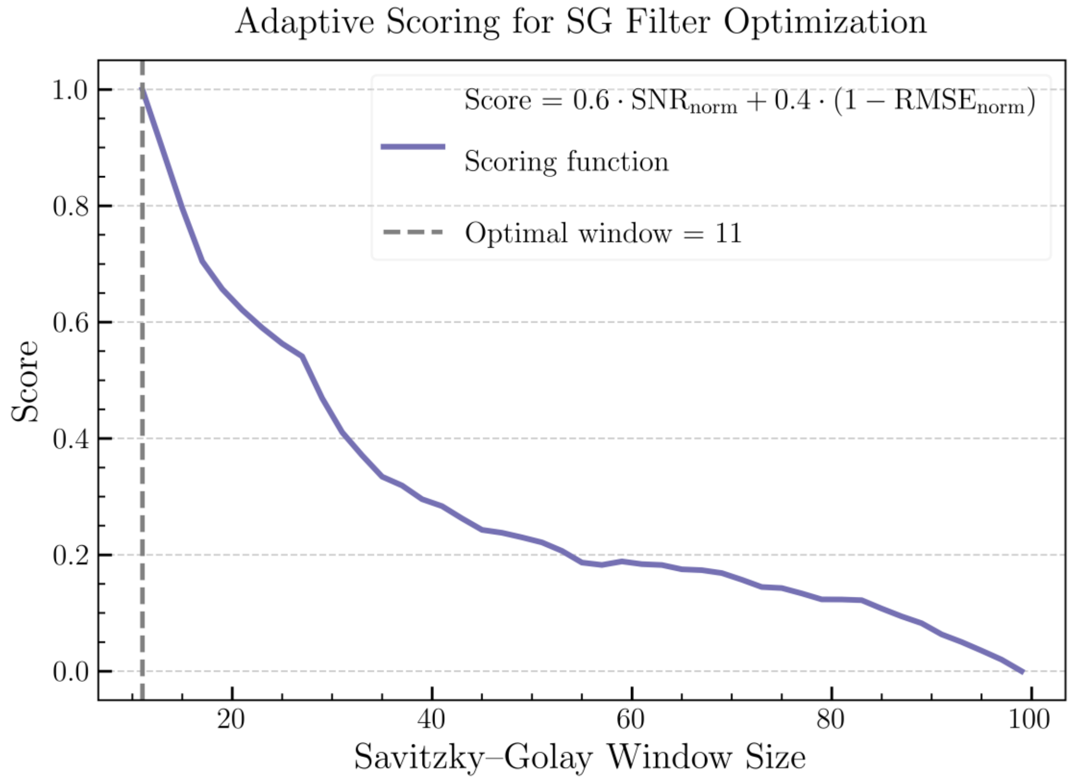

Figure S22. Effect of SG filter window size on SNR and RMSE for Sample M_2, used to determine the optimal adaptive smoothing configuration.

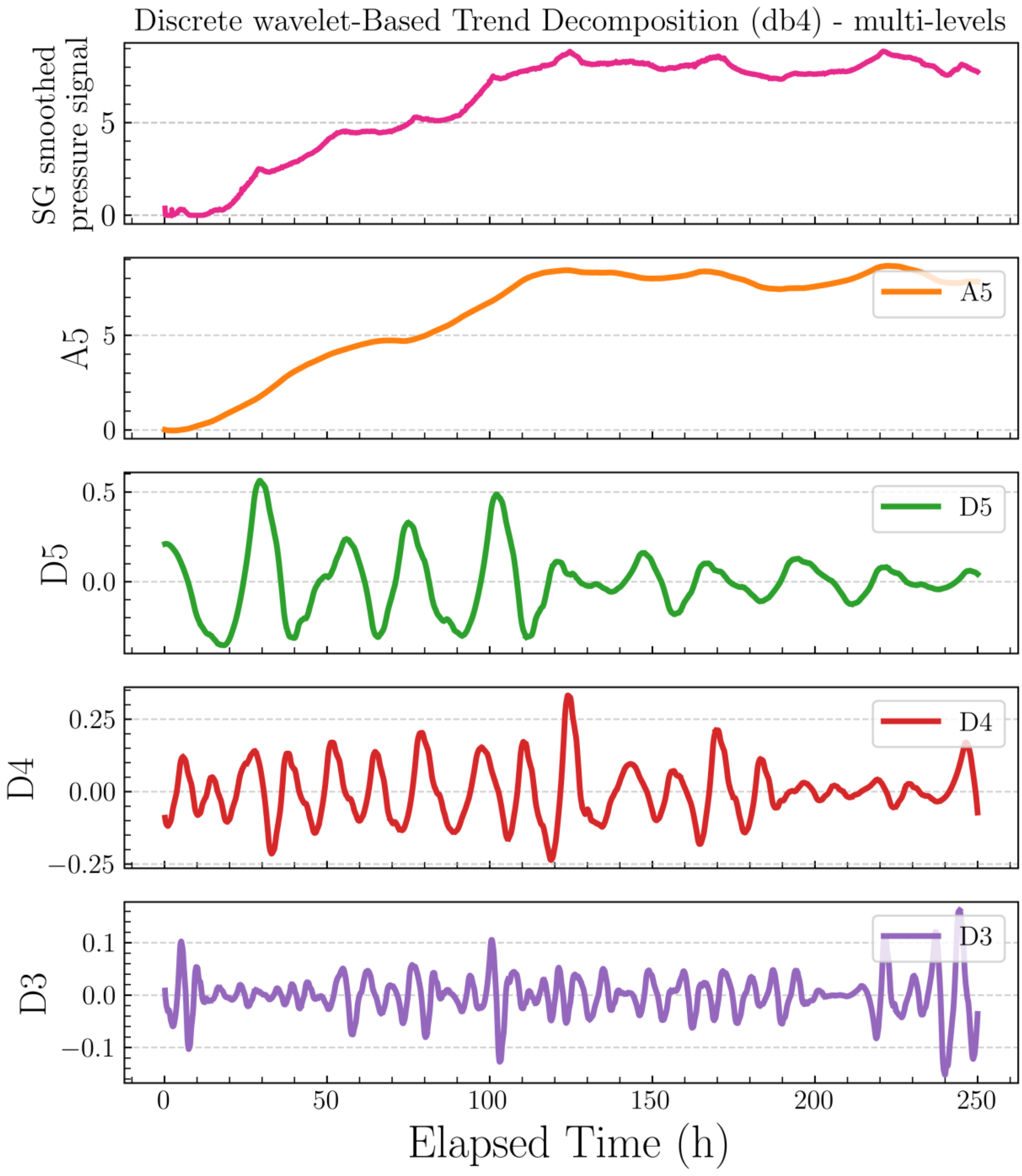

Figure S23. Wavelet decomposition on the SG smoothed data for Sample M_1 at room temperature using DWT (db4), highlighting mid-frequency components capturing the active pressure rise. Only D3–D5 were retained during signal reconstruction due to their dynamic nature.

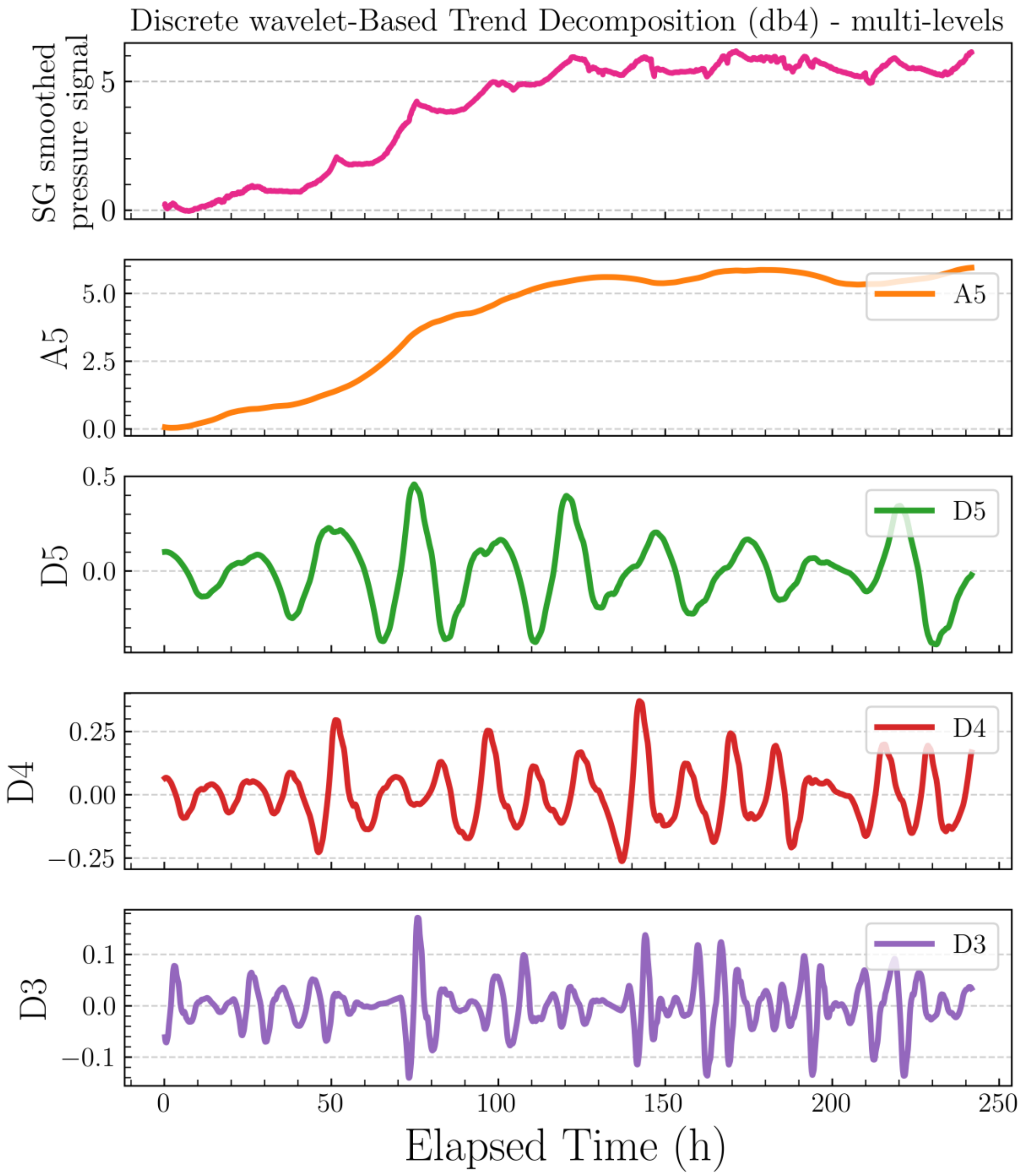

Figure S24. Wavelet decomposition on the SG smoothed data for Sample M_2 at room temperature using DWT (db4). The analysis shows the signal contributions from A5 and D3–D5 levels from low-to-mid frequency components.

Table S6. Supplementary kinetic model fitting results for temperature dependent behavior based on Arrhenius-type analysis at room temperature (RT).

Sample	T (°C)	Model	RMSE	AIC	R^2
M_1	RT	logistic	0.475	-1.33e+06	0.967
		first-order	0.669	-7.2e+06	0.936
M_2	RT	logistic	0.173	-3.04e+06	0.993
		first-order	0.569	-9.79e+05	0.922
